# Capturing Dynamics of Biased Attention: Are New Attention Variability Measures the Way Forward?

**DOI:** 10.1371/journal.pone.0166600

**Published:** 2016-11-22

**Authors:** Anne-Wil Kruijt, Andy P. Field, Elaine Fox

**Affiliations:** 1 Department of Experimental Psychology, University of Oxford, Oxford, United Kingdom; 2 School of Psychology, University of Sussex, Sussex, United Kingdom; Ohio State University, UNITED STATES

## Abstract

**Background:**

New indices, calculated on data from the widely used Dot Probe Task, were recently proposed to capture variability in biased attention allocation. We observed that it remains unclear which data pattern is meant to be indicative of dynamic bias and thus to be captured by these indices. Moreover, we hypothesized that the new indices are sensitive to *SD* differences at the response time (RT) level in the absence of bias.

**Method:**

Randomly generated datasets were analyzed to assess properties of the Attention Bias Variability (ABV) and Trial Level Bias Score (TL-BS) indices. Sensitivity to creating differences in 1) RT standard deviation, 2) mean RT, and 3) bias magnitude were assessed. In addition, two possible definitions of dynamic attention bias were explored by creating differences in 4) frequency of bias switching, and 5) bias magnitude in the presence of constant switching.

**Results:**

ABV and TL-BS indices were found highly sensitive to increasing *SD* at the response time level, insensitive to increasing bias, linearly sensitive to increasing bias magnitude in the presence of bias switches, and non-linearly sensitive to increasing the frequency of bias switches. The ABV index was also found responsive to increasing mean response times in the absence of bias.

**Conclusion:**

Recently proposed DPT derived variability indices cannot uncouple measurement error from bias variability. Significant group differences may be observed even if there is no bias present in any individual dataset. This renders the new indices in their current form unfit for empirical purposes. Our discussion focuses on fostering debate and ideas for new research to validate the potentially very important notion of biased attention being dynamic.

## Introduction

Recently, two research groups independently proposed and tested new methods to index individuals’ tendencies to switch their focus of attention towards and/or away from visual emotional information [1,2]. Adding new analysis options for Dot Probe Task (DPT) derived data, these new indices are meant to capture differential dynamics of attention allocation over time.

The dot probe task (DPT) was introduced in 1986 [3] as an alternative to the Stroop task for indexing biased attention allocation. During a single DPT trial, a participant is presented with two visual stimuli simultaneously, typically consisting of an emotional stimulus (e.g. a threatening word or picture) and a neutral stimulus. Upon offset of the stimulus display, a small target appears in the spatial location previously occupied by either the emotional stimulus (in a congruent trial) or the neutral stimulus (in an incongruent trial). The participant is instructed to respond to this target as quickly as possible. The basic premise of the DPT is that if an individual has a tendency to orient visual attention towards emotional rather than neutral information, this tendency (bias) will enable faster responding on congruent trials (when the target appears in the spatial location previously occupied by the emotional stimulus) relative to the incongruent trials. Thus, the difference in response time (RT) for congruent and incongruent trials constitutes the traditional DPT derived bias index (BI). BI is usually assessed over at least 86 trials. Using the DPT, and variations thereof, attention allocation biases have been observed in various patient- and at-risk samples, supporting cognitive theories of psychopathology, which state that (content-specific) attention allocation biases play a role in the aetiology and maintenance of various types of psychopathology [4–7].

Interest in the DPT has surged in recent years, which we putatively associate with the development of DPT variations intended to modify, rather than assess, attention allocation bias. This application of DPT (and similar modifications of other bias assessment tasks such as emotional visual search and single cueing tasks [8,9]) is termed Attention Bias Modification (ABM). Initially proposed as an experimental tool for studying the putative causal role of attention allocation bias in the aetiology of psychopathology [10], its potential as a new treatment modality was soon identified [11]. Especially in the past 6 years an exponential increase in both numbers and scope of DPT based papers can be observed (see S1 Fig). An extensive literature search in the Scopus database yielded 751 empirical papers reporting on dot probe type data since 1986. Of these, 43 were published between 1986 and 2001, 9 in 2002, 44 in 2009, and 117 in 2015. Not only the numbers of papers have increased, DPT based methods are applied to an increasing number of phenomena throughout the spectrum of psychology subfields, ranging from primate research, imaging and physiological studies, through biases associated with clinical disorders and medical outcomes, functional biases in professional contexts (police, safety related bias), to biases related to political views, customer satisfaction, and experiencing nature. A proportion of these papers appear to aim to measure whether attention allocation bias exists in association with phenomena of interest, to assess whether it would be worth pursuing modification of bias.

Yet, the ABM field seems affected by the so-called decline effect: large effects sizes reported in early ABM papers (2009 to 2012) focussing on mood disorders, have not always been replicated and an increasing number of null results become published, often in the context of larger scale RCTs in patient samples (meta-analyses: [12–15]).

The DPT plays two central roles in ABM studies. The most widely tested ABM procedure is in itself a modified DPT [10,16]: and since the DPT is also the ‘gold standard’ for assessing attention allocation bias, it is the logical first choice for assessing near transfer effects of ABM (i.e. effects on bias, rather than effects on symptoms, which we consider far transfer effects). If DPT based ABM procedures do not modify bias as assessed with a procedure nearly identical to the training procedure, this hampers the credibility of ABM’s proposed mechanism of action. Unfortunately, such near transfer is regularly not observed in ABM studies [17]. With the promise of ABM as a treatment and given the current state of affairs, interest in understanding and improving DPT and related methods has grown stronger than ever before, as evidenced by an increasing number of papers focussing on the psychometric properties of the DPT [18–25].

These papers paint a grim picture however, with reliability indices for bias scores typically found to be unacceptably low [18–25]. It is important to realize that there are distributions at two different levels at play. DPT data consists of RTs observed for congruent and incongruent trials, which have a distribution (e.g. a mean and *SD*), yet the outcome of interest is the difference in RT between incongruent and congruent trials, which theoretically reflects bias and has its own distribution (mean and *SD*). In two of the recent psychometric papers, reliability coefficients are given for RTs as well as for bias scores [18,22]. Both these papers report acceptable to good reliability at the RT level, paired with virtually zero reliability for the bias indices (e.g. Spearman Brown corrected split half reliabilities of 0.91 & 0.93 for RT incongruent and congruent, paired with an overall bias index reliability of -0.12 as reported by Waechter and colleagues [22]).

An exciting development therefore, has been the introduction of new ways of thinking about, and interpreting the data that is derived from, the DPT. This includes the presentation of new indices to calculate from DPT data in order to capture the dynamic and highly variable nature of attention allocation, rather than assessing bias as a static phenomenon. These would allow the notion that an anxious individual may regularly switch their attention between negative and otherwise valenced information, which may occur even if a putative dominant tendency to attend towards negative information is present.

**Fig 1:**
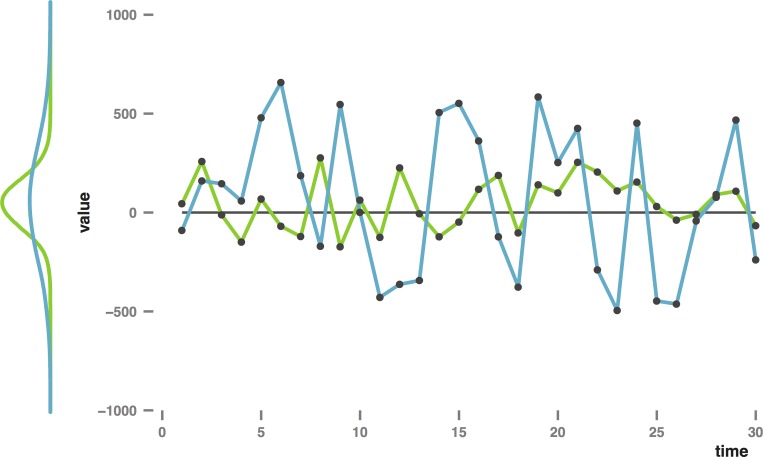
Normally distributed data show an oscillating pattern when plotted over time. Green (light): mean = 0, *SD* = 150. Blue (dark): mean = 0, *SD* = 350. Density plots (left hand panel) based on 10 000 randomly generated data points with a standard normal distribution (using Rnorm()). The lines (right hand panel) show the first 30 data points of each dataset.

The two recently proposed algorithms for analysing DPT derived data are termed Attention Bias Variability (ABV—by Iacoviello and colleauges [2]), and Trial Level Bias Scores (TL-BS–by Zvielli and colleagues [1]). Both seminal papers note that the traditional BI represents a relatively static measure, providing one summary index to represent attention orientation over an entire assessment session of 60 up to several hundred trials. The authors of the new indices propose that “AB (*attentional bias)* may be expressed in fluctuating, phasic bursts, toward *or* away from target stimuli” ([1], p3), and “Therefore, attention-bias variability, or within-subject variability of attention biases toward and away from threat during attention-bias assessment, might explain the seemingly conflicting findings of studies reporting biases toward, and others reporting biases away from, threat-cues in PTSD” ([2], p233). Thus, both TL-BS and ABV indices are designed to reflect the temporal dynamics of attention allocation. To this end, both methods construct a series of bias indices computed over consecutive subsets (ABV) or pairs (TL-BS) of DPT trials. The concept of attention variability seems best explained using a graphical representation similar to Fig 1: a time series of bias indices computed over pairs of incongruent and congruent trials, which will typically exhibit a cyclic pattern. This is proposed to reflect attention variability, with positive values indicating attention oriented towards the emotional stimulus (relative to the neutral stimulus) and negative values reflecting attention oriented away from the emotional stimulus. Thus, the cycling pattern is interpreted to reflect an individual’s attention moving back and forth over time, towards and away from the emotional stimulus. Moreover, differential patterns were observed between patient (spider phobia, PTSD) and control groups, and between smokers and non-smoking controls, such that the patient and smoker groups showed cyclic patterns with larger amplitudes. The authors proposed that these reflect higher variability in attention allocation in these groups, relative to a more stable pattern of attention orienting in control groups [1,2]

Importantly, bias time series, similar in appearance to Fig 1, are not the main outcome of the new variability methods. The bias time series are used to derive summary indices. In the ABV method [2], the time series is constructed by calculating BI for each of eight consecutive ‘bins’ of 20 trials. To arrive at the ABV index, the standard deviation of these eight BI is divided by the overall mean RT. In the TL.BS method [1] every single congruent and incongruent trial is paired with the opposing (incongruent/congruent) trial that occurred closest in time, constrained at a maximum distance of five trials back-, and forward. For each trial pair, a BI is calculated (RT incongruent minus RT congruent). The resulting series of TL-BSs (Trial Level Bias Scores) is then used to derive five indices. Peak and average values are analysed separately for positive and negative TL-BSs. The fifth index, TL-BS variability, is calculated as the mean absolute distance over the entire series of TL-BSs. Thus, both methods take the mechanics of the DPT into account, assuming that RT differences between the two trial types (incongruent–congruent) reflect preferential attention allocation yet add the notion that these RT differences may vary rapidly and meaningfully over time.

Although very similar to graphical representations of observed bias dynamics over the course of a DPT assessment (compare Fig 1 reported by Zvielli and colleauges [1]), Fig 1 does not represent DPT data. The right hand panel shows the first 30 data points from two datasets of random, normally distributed, data, with parameters *M* = 0, and *SD* = 150 and 350 respectively. Density plots for the entire datasets (*n* = 10 000 each) are shown in the left hand panel. The currently presented study is based on a central tenet of classical test theory, namely that any observed value is a combination of a true score plus measurement error. Measurement error usually results in both under- and overestimation of the true score, and thus a collection of observed values typically shows a two-tailed distribution. For any such two-tailed distribution, mapping each data point’s value (*y*) as a function of time (*x*) results in a graph with a cycling pattern: on consecutive draws, data points ‘fall’ randomly above or below the mean value, with most data points falling close to the mean, and a smaller number of data points falling farther away from it. The amplitude of the resulting cyclic pattern corresponds to the standard deviation of the distribution. In most regular statistics, the standard deviation is used as an estimation of the measurement error. Importantly however, in the context of repeated measurements a high standard deviation in data obtained from a single individual may reflect that either the construct being measured is unstable (high variability), that the measurement method itself is unreliable (high measurement error), or a combination of both.

The new DPT indices, therefore, likely provide an index of attention bias variability and measurement error combined. This paper presents the results of a series of simulations devised to assess the relative sensitivity of these measures to changes in measurement reliability in the absence of attention bias, and changes in attention bias variability in the absence of measurement reliability changes. We propose that in Dot Probe data (variability in) attention bias can only be reflected in (variability in) RT differences between incongruent and congruent trials. If however, the RT for congruent and incongruent trials are sampled from the same distribution (i.e. same mean and *SD*), there can be no bias according to the inherent logic of the DPT. Therefore, variability at the RT level observed in a dataset in which the RT for congruent and incongruent trials are interchangeable in terms of their underlying distributions, may be regarded as reflecting mostly measurement error. Therefore, the new measures should ideally respond more strongly to variability in bias (a difference between IT and CT distributions) than to variability (*SD*) in RT for both congruent and incongruent trials in the absence of attention bias.

To address the question of relative sensitivity to bias variability versus measurement error, series of randomly generated datasets with DPT characteristics were processed according to either the ABV or TL-BS, and the traditional BI methods. For both new methods, three initial simulation series were run, each systematically manipulating a single parameter of the standard normal distributions of randomly drawn ‘raw RT’ data. These simulations provide insight into the extent to which traditional BI and the new variability measures are sensitive to 1) increasing standard deviation at the RT level (in the absence of attention bias), 2) increasing mean RT (in the absence of attention bias), and 3) increasing attention bias (i.e. increasing the mean RT *difference* between congruent and incongruent trials).

To assess sensitivity to variability in biased attention over time, we then proceed with two additional series of simulations aimed at either one of two characteristics of variability. Given the cyclic patterns, variability can be defined by two characteristics. Either attention bias may switch less or more often (i.e. the frequency of bias switches may vary), or bias may be more or less pronounced (i.e. the magnitude of attention bias *in the presence of bias switches* may vary). If individuals indeed differ in the variability of attention bias, these differences will most likely be best described as a combination of both frequency and magnitude differences. However, for the current purpose of assessing which data characteristics are reflected by the new variability indices, these characteristics are independently manipulated in separate simulations series. These use datasets in which an attention bias is implied and is made to switch from a positive to a negative value and vice-versa. In the first type of these dynamic simulations a frequency difference is created while bias magnitude is kept constant. In the second type of dynamic simulation a bias magnitude difference is created while sign-switching frequency is kept constant.

We hypothesize that ABV and TL-BS indices will respond to increases in *SD* at the RT level. In addition, ABV is expected to respond to increases in overall mean RT (due to this being the denominator of the ABV formula). Traditional BI is expected to selectively respond to increased difference between the mean RT for congruent and incongruent trials. The two dynamic simulations are more exploratory in nature, we do not formulate specific hypotheses for the responses by the new indices for these simulations. Traditional BI is expected to respond to the presence of one or more BI switches, representing the average bias implied within each of the individual simulated datasets.

## Methods

### General Methodology

All simulations and analyses were performed in R version 3.1.2 (“pumpkin helmet”). All simulation scripts and aggregated data files are available from https://figshare.com/articles/Capturing_Dynamics_of_Biased_Attention/1515002 (DOI: 10.6084/m9.figshare.1515002)

Random data were generated using the *rnorm()* function, which allows specification of the number of data points to be generated as well as the mean and *SD* of the distribution from which these data are drawn. Data were generated separately for each type of trial (congruent (CT), incongruent (IT), and neutral-neutral (NT) trials) for each ‘participant’ *i*. Three data subsets (CT/IT/NT) were then randomized into one ‘*i*’ dataset to represent the random order of trial types in a single DPT assessment. The start values in the simulations assessing effects of increasing mean, *SD*, or traditional BI were set at *M* = 600, *SD* = 30. In the increasing mean and *SD* simulations, the same *M* and *SD* were used to generate IT and CT trials within each dataset. Thus, no bias (IT-CT difference) exists in these datasets. Within each simulation increases in *SD*, means, bias, or bias dynamics were created by manipulating the settings for datasets ‘*i*’ assigned to the change groups (‘A’), while the settings for datasets for the control groups (‘B’) retained their start values.

#### Increasing mean simulations

For each subsequent run (‘r’) of 1000 studies, mean values for both CT and IT trials in the change groups were increased with 20 units (‘milliseconds’), from *M* = 600 in run 1, to *M* = 620 in run 2, up to *M* = 780 in run 10.

#### Increasing *SD* simulations

For each subsequent run (‘r’) of 1000 studies, the *SD* value for both CT and IT trials in the change groups was increased with 2 units, from *SD* = 30 in run 1, *SD* = 32 in run 2, up to *SD* = 48 in run 10.

#### Increasing bias simulations

For each subsequent run (‘r’) of 1000 studies, the mean RT value for CT trials was decreased with 1.5 units, and the mean RT value for IT trials increased with 1.5 units. Thus a ‘RT difference’ (bias) was created and increased with 3 units on each consecutive run: bias = 0 in run 1, bias = 3 in run 2, up to bias = 27 in run 10.

#### Increasing dynamic frequency simulations

For the simulations assessing effect of increasing the frequency of BI switches, bias magnitude (IT-CT difference) was kept constant at +20 in datasets in the control groups. In the change groups an increasing number of switches were created over consecutive runs ‘r’, such that bias changed sign from +20 to -20 and vice versa. On each run ‘r’, bias switches were implemented to occur following every ‘1/r’th trial. Thus no switch was present in run 1 (a constant bias of +20 was implied), one switch was created after 1/2 the trials in run 2, up to switches occurring every 1/10^th^ of the trials in run 10. Data were generated (i.e. *rnorm*() called) for all trials that share the same characteristics at once, after which the generated random values were relocated to a randomized order of CT/IT/NT trials. I.e. in run 4 there were 3 switches and therefore 4 ‘data sub sets’ (with bias +20, -20, +20, and -20 respectively). All IT trials with *M* = 610 were generated in one call of the *rnorm*() function, and all IT trials with *M* = 590 in another, after which these values were allocated to trial numbers randomly assigned to represent IT trials in their respective subsets. In some runs, the total number of trials cannot be divided by ‘r’. In their datasets ‘i’, leftover trials were added to the last subset. For instance, when 160 trials were divided by 3 (in run 3 for the ABV method in which 2 switches were implemented), switches were created following every 53 trials, i.e. following trial # 53, 106, and 159. This results in three subsets of 53 trials, plus one leftover trial, which was added to (i.e. assigned the same characteristics as) the third subset. Notice that this does not necessarily render the last subset different from the other subsets, as these will also vary slightly in their number of CT, IT, and NT trials due to the trial order being randomized independent from the number of switches as well as due to outlier trials being removed (see below). Also notice that for the TL-BS methodology, the number of CT and IT trials was 20 each (see explanation below). Therefore, in both runs 7 and 8, switches were created following every 5^th^ trial, and in runs 9 and 10 following every 4^th^ trial, rendering these two sets of runs functionally similar.

#### Increasing dynamic magnitude simulations

For the simulations assessing effects of increasing bias amplitude in the presence of dynamic bias, datasets for both control and change groups were constructed to always have three bias switches (e.g. four subsets with bias is +20, -20, +20, -20). Bias magnitude (IT-CT difference) was set to increase by 3 points on each consecutive run ‘r’ (e.g. +23, -23, +23, -23 in run 2, up to +47, -47, +47, -47 in run 10) in the change groups. Bias magnitude was kept constant at +/- 20 in datasets in the control groups.

Once generated, each dataset ‘i’ was processed according to either the ABV or TL-BS methods.

### ABV methods

In line with study 1 reported by Iacoviello et al (2014), each dataset ‘i’ consists of 64 incongruent trials, 64 congruent trials, and 32 neutral-neutral trials in random order. Although there were no trials on which ‘participants’ made an error to discard, RT values < 150 and > 2000, and all values deviating more than 2 *SD* from the individual mean (computed separately for CT, IT, and NT trials) were discarded, in line with the rules applied by Iacoviello and colleagues [2]. The remaining data were divided into bins of 20 original trials (i.e. trial number 1–20, 21–40, etc., regardless of the number of discarded trials within each bin). Bias index (BI) was computed for each of the eight bins as ‘ mean RT(IT)−mean RT_CT)_ ‘, after which the *SD* for the eight bin BIs was obtained. ABV was computed as ‘*SD*_(BI across bins)_ / mean RT_(CT+IT)_’. On rare occasions the random order of IT/CT/NT trials resulted in no CT or IT trials being assigned to at least one of the eight bins. This occurred between 163 and 186 times per run of 520 000 datasets (i.e. in .031 - .036% of datasets). We decided to not alter the ABV formula for these datasets (for instance by using 7 bins rather than 8), but to retain the value *NA* returned for these data sets’ ABV computation.

### TL-BS methods

Following the methods of study 1 reported by Zvielli and colleagues [1] each dataset ‘i’ consists of 20 congruent, 20 incongruent, and 20 neutral-neutral trials. For these analyses outliers were defined according to the rules used by Zvielli et al. such that RT values < 200 and > 1500, as well as data points deviating more than 3 *SD* from the individual RT mean were discarded. The resulting data for CT and IT trials were then paired for computing TL-BS. Each CT and IT trial was paired to its temporally most contiguous opposite trial, with a maximum of five trials distance in either direction. For each trial pair, a TL-BS was calculated as ‘RT(IT-trial)−RT_(CT-trial)_’. Peak and average values of positively and negatively valued TL-BSs were logged, as well as the TL-BS variability parameter computed as the sum of absolute differences between each consecutive TL-BS divided by the total number of TL-BSs (Zvielli, et al, 2014). Upon learning that TL-BS variability is meant to be calculated as the total length of the TL-BS lines using the Euclidian distance formula (Zvielli, Bernstein, Koster, personal communication), we adjusted our scripts. All TL-BS variability indices reported were calculated as ‘ mean (sqrt ((trials distance between 2 consecutive TL-BS)^2 + (value difference between 2 consecutive TL-BS)^2)) ) ‘. We noticed that increasing bias also increases the probability of no negative TL-BS parameters being generated. I.e.: as the mean RT for IT trials increases relative to the mean RT for CT trials, the probability of ‘ RT(IT-trial)−RT_CT-trial)_’ for any trial pair resulting in a negative value decreases. This situation occurs almost exclusively in simulations wherein a bias difference is implied, i.e. 82 occurrences among 520 000 datasets ‘i’ (.016%) in the increasing bias simulation and 92 in the increasing dynamic bias frequency simulations (.018%), versus 0 occurrences in the *SD* and mean increasing simulations. The bias increased simulation also had a single occurrence of an individual dataset in which no positive TL-BSs were generated. When no negative or positive TL-BS were generated for a single dataset ‘i’, the values for average and peak negative/positive TL-BS were set at ‘0’, which we argue is a meaningful value in this context.

### Outcome Measure

In order to provide a demonstration of how the measures would perform in studies assessing group differences using these indices, group differences within each ‘study’ dataset *j* (consisting of two groups of 26 ‘participant’s *i*) were assessed using Welch *t*-tests. Welch’s *t*-test is the default *t*-test in R, yet in the context of, especially, the *SD* increasing simulations it would be the *t*-test of choice as it is more robust against unequal variances. For every run of 1000 studies, the percentage of significant *p*-values (*p* < .05) was obtained. Given an alpha of .05, among 1000 *t*-tests, circa fifty (5 percent) false-positive *t*-tests are expected to occur in the absence of a ‘real’ difference. Increasing percentages of significant *t*-tests over runs within in a simulation series, indicate the measure’s sensitivity to the change being implemented. Sample size (*n* = 26 per group) was chosen such that *t*-tests have 80% power to detect at least large effect sizes (*d* = .80). Therefore, each individual ‘study’ *j* is underpowered for detecting small or moderately sized effects, rendering our outcome of percentage significant *t*-tests relatively conservative. Ideally (yet contrary to our hypotheses), the percentage of significant *t*-tests for the new DPT derived bias measures will increase selectively in either one or both of the bias variability simulations, while remaining stable at 5% in simulations wherein no bias is created (i.e. the *SD* and mean RT increasing simulations in which congruent and incongruent trials have identical characteristics within each dataset *i*).

### Procedure

All reported data were generated in a single consecutive run of eight simulations *s*, each generating data for 10 runs *r*. Each run contains 1000 studies *j*, and each study *j* contains 52 datasets *i* assigned to either change or control groups. For each study *j*, Welch’s *t*-test was performed to test for differences between change and control groups (each containing 26 datasets *i*) for the ABV or TL-BS parameters, and traditional BI (computed as ‘ mean RT(IT)−mean RT_(CT)_ ‘ for all included trials). For every thousand studies (a single run *r*), the percentage of significant *p*-values (*p* < .05) was obtained. One simulation *s* consists of ten runs *r* in which either *SD* RT, mean RT, bias magnitude, dynamic frequency, or dynamic magnitude of bias increase in the change groups over consecutive runs according to the rules explained above.

## Results

**Fig 2:**
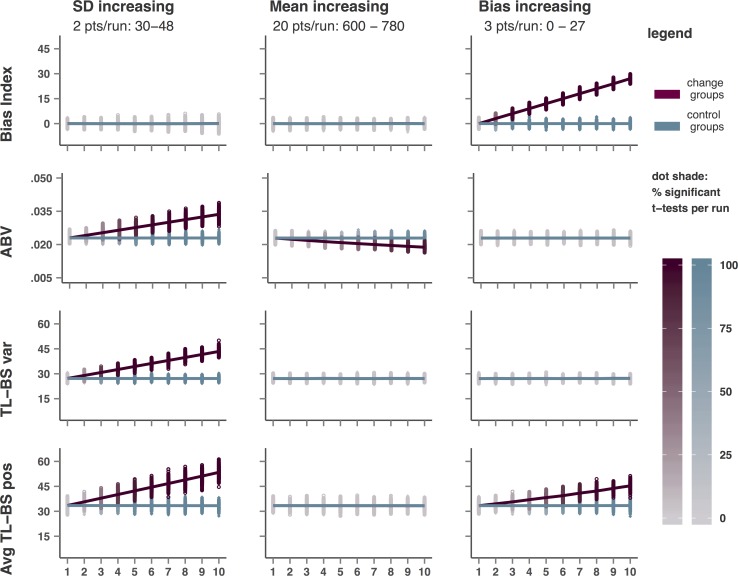
Observed average values of BI, ABV, TL-BS variability, and Average TL-BS positive for each study and each run of the first three simulation series (*SD*, mean, and bias increasing). Each data point represents the group average for a single study, lines represents the average observed for each run of 1000 studies. The shades of the data points (but not the lines) indicate the percentage significant group differences observed per run (1000 studies).

Fig 2 provides a graphical representation of the outcomes for each type of simulation for the traditional BI, ABV, TL-BS variability, and the average TL-BS positive measures. For space reasons, only one of the four TL-BS positive/negative measures is shown here. The results for peak TL-BS indices are more pronounced than for average TL-BS indices, whereas the TL-BS negative indices show a pattern opposite to their positive counterparts. Fig 2 shows the BI outcomes as provided by the ABV simulations. These simulations show slightly more pronounced BI effects compared to the TL-BS simulations, due to being based on more trials (i.e. higher power). The complete set of outcomes is provided in tabular form in S1 Table (ABV) and S2 Table (TL-BS).

In the following paragraphs the results of the ABV and TL-BS simulations will be explored and compared for each type of simulation.

### Increasing RT *SD*

**Fig 3:**
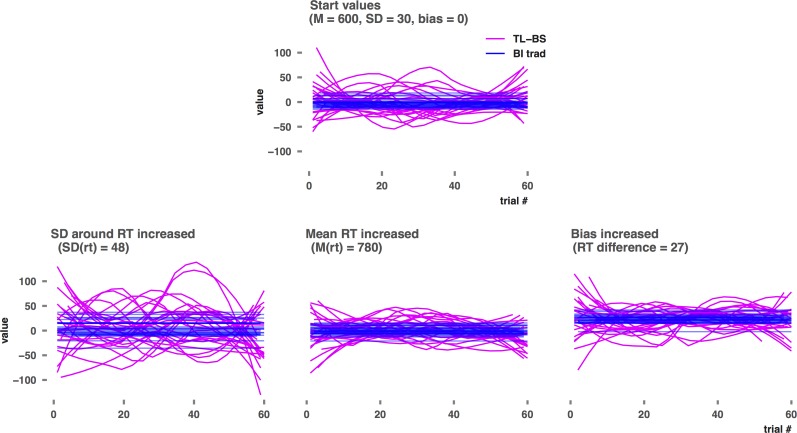
TL-BS time series and traditional BI for increased *SD*, mean, and bias. Panels B, C, and D show data for the 'change group' in the 1000th study of the 10th run in the increasing *SD*, mean, and BI simulations respectively. Similar to Fig 2 reported by Zvielli and colleauges [1], a smoothing procedure was applied to the TL-BS data.

As hypothesized both ABV and TLBS indices respond strongly to an increase in *SD*. Even at *SD* differences as small as 2 points (30 vs 32 in run 2) *t*-tests indicate significant group difference for the dynamic measures in 13.5% (ABV) up to 27.8% (TL-BS variability) of the studies *j*. Percentage significant *t*-tests for group differences maxes out at 100% from *SD* differences of 8 points for TL-BS variability, 14 points (peak TL-BS negative) or 16 points (for the remaining indices) onwards (see S1 Table and S2 Table). Note that the characteristics (*M* and *SD*) of the distributions from which the CT and IT RT values were drawn, were identical within each individual dataset *i*. Thus, significant *t*-tests cannot indicate the presence of bias or variability thereof. For the traditional BI measure the number of false positive *t*-tests is stable and corresponds to an alpha of .05. Comparing Fig 3 panel B to panel A shows how *SD* differences will result in significant group differences for the positive and negative peak and average TL-BS indices. These indices represent actual TL-BS values and thus increase as the amplitude for the TL-BS pattern increases. The TL-BS variability index, on the other hand, becomes significant due to the longer TL-BS lines in panel B relative to panel A. To understand the results for the ABV measure, it suffices to consider that increasing *SD* increases the numerator of the ABV formula (*SD*_(BI across bins)_ / mean rt_(CT+IT)_).

### Increasing Mean RT

As hypothesized, ABV responds slightly to increasing differences in overall mean rt. ABV is clearly affected from run 3 onwards in which a mean RT group difference of 40 ms results in 13.9% significant *t*-tests, In run 10 (180 ms RT difference) the ABV index indicates significant group differences in 91.1% of the studies, despite the differences between mean RT for congruent and incongruent trials within each dataset being zero. This sensitivity in the absence of bias can again be understood from the ABV formula, in which the overall mean RT is the denominator: as mean RT increases, the ABV index decreases. TL-BS indices and traditional BI do not respond to increasing differences in mean RT. For each of these indices the percentage of false positive *t*-tests remains at 5%, corresponding to an alpha of .05.

### Increasing bias

For this simulation it is important to note that only the mean difference in RT for IT and CT trials is manipulated, while no difference in variability thereof is implemented. Thus, this simulation does not provide a test of sensitivity to bias variability, but of sensitivity to bias magnitude only. Despite differences in BI up to 27 being implemented in the increasing BI simulation, neither ABV nor TL-BS variability responds to increasing bias magnitude. As expected BI responds strongly to implemented bias. In the TL-BS simulation, BI picks up bias differences of 3 points in 19,0% of the datasets, while bias differences of 15 points or more are detected in 100% of the studies. Due to being based on larger datasets *i*, the BI measure has more power in the ABV simulation, were it detects bias of 3 ‘milliseconds’ in 49.7% percent of the studies and reaches 100% significant t-tests at a bias of 9 milliseconds. The TL-BS positive/negative indices also show sensitivity to increasing bias magnitude, although less than traditional BI. They return between 90.3% (average TL-BS negative) and 99.6% (average TL-BS positive) significant *t*-tests at a bias difference of 27 points in run 10. This difference ties in with findings by Zvielli and colleagues, who reported better prediction of daily smoking rates for the TL-BS positivity indices compared to the TL-BS negative indices. We suggest this to be due to less negative (and more positive) TL-BSs being generated in the presence of a (positive) bias, when for any trial pair the IT-CT difference (i.e. TL-BS) is more likely to take a positive value. Comparing Fig 3 panel D to panel A illustrates that, similar to BI, the TL-BS data pattern is elevated in its entirety, but does not intrinsically change as bias increases. This explains why TL-BS variability does not respond to increasing bias magnitude, as it reflects relative distances between the TL-BS rather than actual values.

### Increasing dynamic bias frequency

**Fig 4:**
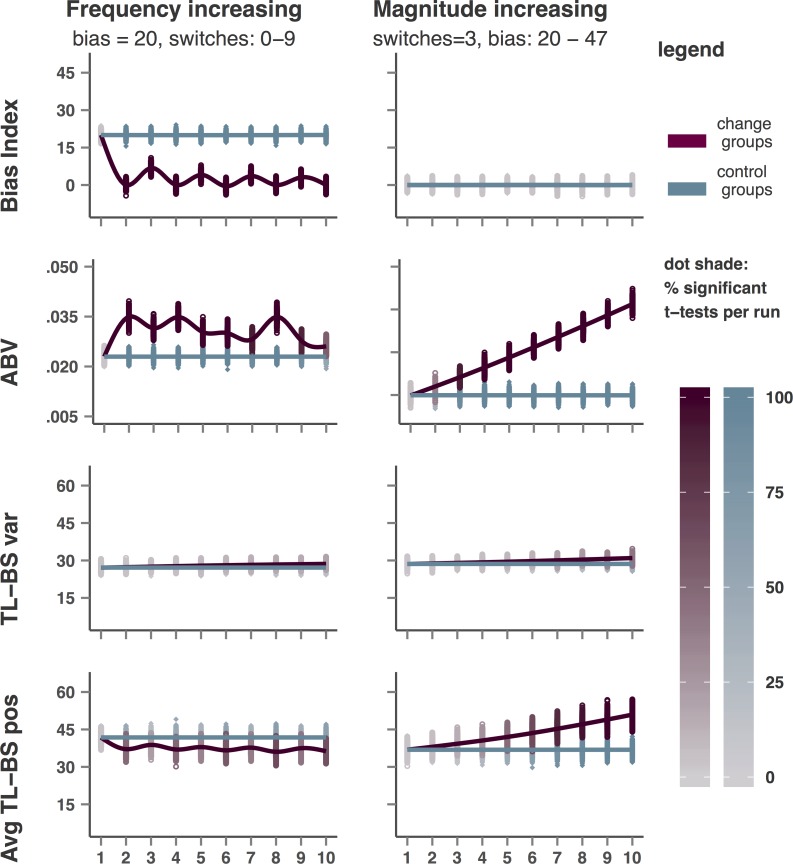
Observed average values of BI, ABV, TL-BS variability, and Average TL-BS positive for each study and each run of the dynamic bias simulations. Each data point represents the group average for a single study, lines represents the average observed for each run of 1000 studies. The shades of the data points (but not the lines) indicate the percentage significant group differences observed per run (1000 studies).

**Fig 5:**
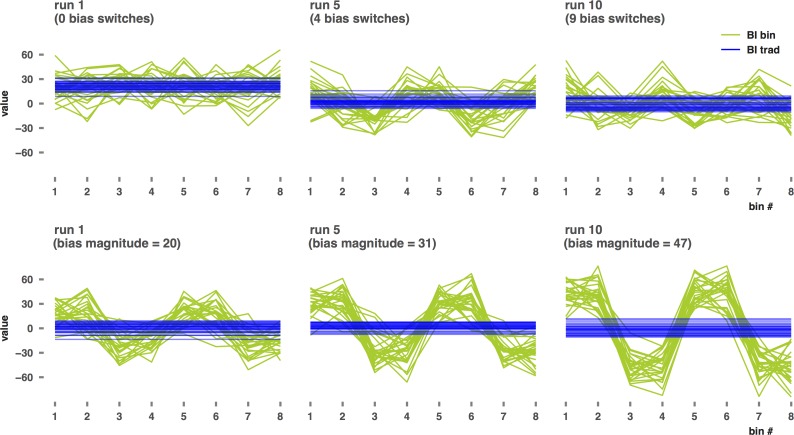
ABV time series (BI per bin) and traditional BI when increasing dynamic bias switching frequency and increasing dynamic bias magnitude. Each row depicts data for the ‘change group’ in the 1000th study of runs 1, 5, and 10 of the increasing dynamic frequency simulation (top) and the increasing dynamic magnitude simulation (bottom).

Fig 4 shows the results for BI, ABV, TL-BS variability, and average TL-BS positive for each of the two dynamic simulations. In the first of the two dynamic simulations, BI was kept at +20 in datasets for control groups, whereas an increasing number of BI switches between +20 and -20 were created within datasets in the change groups. To illustrate these simulations’ mechanics, the top row of Fig 5 shows observed BI and the ABV time series for the change group in study 1000 of runs 1, 5, and 10, in which 0, 4, and 9 switches were implemented respectively. Traditional BI detects the presence of either one or more bias switches, but does not respond linearly to an increasing number of switches. BI moving up and down in runs 2 to 10 of this simulation (Fig 4) can be explained from the proportion of trials with a positive or negative bias implied. In runs with an odd number of switches (i.e. runs 2, 4, 6, 8, and 10) a negative bias (- 20) was implied in half of the trials while a positive bias (+20) was implied in the other half, rendering the overall BI zero. In runs with an even number of switches, a smaller proportion of trials had a positive bias difference implied (i.e. one third in run 3, two fifth in run 5, etc.), rendering the overall BI value at one thirds between -20 and +20 in run 3, at two fifths between -20 and +20 in run 5, etc. Given the sensitivity of the TL-BS positive/negative indices to bias changes, as demonstrated in the bias increasing simulation, this same explanation holds for the alternating pattern observed for these indices. To explain the alternating pattern for the ABV index the alignment of each of the eight trial bins with the bias switches has to be taken into account. In aligned runs (2, 4, and 8) the observed BI within each bin is either high (+20), or low (-20). In runs wherein bias switches occur within bins, the BI calculated for these bins deviates less from 0, resulting in a lower *SD* of BIs over the eight bins and thus a lower ABV value. Finally, TL-BS variability does respond slightly to increasing frequency of bias switches and does so in a linear fashion as well. Yet, at 9 bias switches (which in the TL-BS datasets means that BI switches sign after every 2 IT and 2 CT trials on average) significance is achieved for only 19.6% of the group comparisons at study level.

### Increasing dynamic bias magnitude

Finally, the results of the increasing dynamic magnitude simulation are shown at the right-hand side of Fig 4, while the mechanics of this simulation are illustrated in the bottom panel of Fig 5. In this simulation ABV as well as the TL-BS indices respond in a linear fashion, demonstrating their sensitivity to differences in amplitude in bias *in the presence of bias switches*. Notably, the ABV index responds strongly to difference in bias magnitude in the presence of switches (with 37.5% significant *t*-tests in run 2, and maxing out at 100% significant group differences in run 5) while not responding to similar differences in bias magnitude in the absence of bias switches. The TL-BS positive/negative indices respond to a similar extent as in the bias increasing simulation, and less strongly than for the *SD* increasing simulation. For these measures 92.6–99.8% significant *t*-tests were observed in run 10. The TL-BS variability index, finally, responds slightly more strongly in the dynamic magnitude increasing simulation than in the dynamic frequency increasing simulation, but not nearly as strong as in the *SD* increasing simulation. At run 10 (bias: +47, - 47, +47, - 47) the TL-BS variability index indicates significant group differences in 42.7% of the studies. Traditional BI did not respond in the dynamic magnitude increasing simulation but remained stable around value 0 (with 5 percent false positive t-tests), which is the resulting value of equal positive and negative bias subsets within each dataset. Note that if we had defined an even number of switches (resulting in an odd number of subsets), the resulting value for traditional BI would have shifted, as explained for the dynamic frequency simulation above.

## Discussion

Analyzing randomly generated data, a series of simulations demonstrate that newly proposed indices of dynamic attention allocation are all sensitive to difference in *SD* at the RT level in the absence of bias (i.e. when the mean and *SD* of the distributions from which the RTs for the two trial types are drawn, are identical within each ‘individual level’ dataset). In addition the ABV measure is also found to be sensitive to the changes in overall mean RT, again even when the RT for congruent and incongruent trials are sampled from identical distributions. Neither ABV nor TL-BS indices were found to be responsive to changes in the magnitude of bias in the absence of bias variability. In two bias dynamics simulations, ABV and the TL-BS positive/negative indices respond to increasing frequency of switches in attention allocation towards or away from the emotional stimulus, yet in a non-linear fashion. Finally, each of the new indices respond linearly to increasing magnitude of bias in the presence of bias switching between positive and negative values (i.e. towards and away from valenced information).

Importantly, sensitivity to changes in RT mean (ABV) and *SD* (ABV & TL-BS indices) were observed even when the RT for congruent and incongruent trials (within individual datasets *i*) were sampled from the same distributions and therefore should be regarded interchangeable. The inherent logic of the DPT dictates that in the absence of IT-CT differences, any observed statistical difference cannot be reflecting attention allocation bias defined as a differential response time for congruent and incongruent trials.

When no bias dynamics were implemented, the new measures either did not respond at all or to a smaller extent than the traditional BI to RT differences between congruent and incongruent trials (i.e. bias). The two dynamic bias simulations show that in the presence of bias dynamics (operationalized as sign switches over subsets of trials) the new measures are more sensitive to magnitude differences than to frequency differences in bias dynamics. Yet, those measures that respond to a satisfactorily extent (ABV and the TL-BS positive/negative indices) respond non-linearly to increases in dynamic bias frequency and also respond strongly to increasing *SD* in the absence of bias. Thus, for any individual study that returns a significant group difference, or even for any two individual values, it cannot be known whether the higher value reflects a higher or lower frequency of bias switching, a difference in magnitude of a dynamic bias, *SD*_(RT)_ differences in the absence of bias, or a combination of these characteristics. Thus, in their current form, the ABV and TL-BS indices are not suitable for empirical purposes.

When comparing the current results to previously reported results obtained from real data, there are two important things to keep in mind. Firstly, our simulated data is particularly ‘tidy’. Within study groups, all ‘participants’ show similar mean RT, *SD*_(RT)_, and/or magnitude of bias, and in the dynamic simulations ‘participants’ all switch attention after the same number of trials. Also, as opposed to what is typical for RT data, our data is not skewed. This provides our analyses with more control over the data characteristics we wish to manipulate, and more power than any real dataset could. Our results provide a ‘proof of principle’, showing the probabilities of measures responding one way or another when certain specified data characteristics are manipulated. Secondly, if our results suggest that for a given set of data characteristics, the alpha is increased from .05 to say .20, that is highly problematic from an empirical point of view, yet for any individual dataset the probability of not finding a false positive difference is still 80%.

For these reasons, it is not a particularly useful exercise to engage in a detailed mapping of our results onto previously reported results [1,2]. Nonetheless, we wish to address the excellent efforts undertaken by Zvielli and colleagues in order to provide a number of cross validating analyses. Their cross-validations include one specifically aimed at the possibility that TL-BS might be affected by variance. ‘Fake’ TL-BS’s (terminology by Zvielli et al.) were computed by randomly assigning a mock CT or IT ‘status’ to the neutral-neutral trials (NT) in each of their datasets. The resulting TL-BS indices were found to be not associated with arachnophobia status (study 1) or daily smoking rate (study 2). For study 1 three factors may be at play, namely reduced power due to only half the number of trials being available, reduced power relative to our simulations due to this being real (and therefore more ‘messy’) data, and finally the possibility that the *SD* for the NT did not differ between the two groups, despite the presence of a mean RT difference (predicting group status) and considerable *SD* differences observed at the bias index level (data characteristics at trial level were not reported). We verified that the use of logistic regression is not a factor here: in our simulation data comparing percentage of significant group differences for logistic regressions versus Welch’s *t*-tests, yields a maximum difference in percentage significant results per run of 5%. In Zvielli et al.’s study 2, traditional BI and the TL-BS positive indices correlated with smoking rate, while the negative TL-BS and the TL-BS variability indices did not. Based on our results, we suggest this to indicate absence of systematic *SD* changes at the RT level along the smoking rate continuum, as such would likely have caused the TL-BS variability index to correlate with smoking rate. This would explain why Zvielli and colleagues found their ‘fake TL-BS’ to not correlate, despite having the same numbers and thus power as the real-TL-BS in study 2. In the absence of *SD* and despite the presence of mean RT differences, the ‘fake TL-BS’ (which based on neutral-neutral trials cannot represent bias) would respond similarly to the TL-BS in our mean increasing simulation, i.e. not at all. The reported associations of daily smoking rate with the real TL-BS positive indices on the other hand, can at least partly be ascribed to the presence of bias. In addition, and as explained in the methods section, in the presence of a positive bias more positive than negative TL-BS are generated, which may explain why, in study 2, the positive TL-BS indices returned significant results and the negative did not. Nonetheless it is indeed possible that the TL-BS measures have responded to higher variability in attention allocation bias in the experimental group in study 2, as underscored by Zvielli and colleagues showing that the TL-BS positive parameters explain variance beyond traditional BI. Yet in study 1, BI and TL-BS positive parameters did not explain variance beyond mean RT differences.

### Future Directions

In spite of the current results, we do not wish to dismiss the notion that attention allocation bias is likely to be a highly dynamic phenomenon and the need to move beyond the traditional static BI. There is a high ecological validity to this notion, which we fully support. However, our current results indicate clearly that, at least in their current form, the two new methods do not provide an adequate assessment of the dynamic nature of biases in attention allocation. It is currently completely unclear which processes determine the obtained values on these indices: bias dynamics, cognitive control type processes or other (random) influences. Given our findings, we feel that it is very worrisome that dynamic bias measures are being used as if they are already fully developed and validated, bypassing the question of what it is that they measure. They are being implemented at a rapid speed, thereby propagating the idea that these methods provide valid and reliable indices of attention bias, suitable for use in clinical samples [23,26–28] and as a target outcome for treatment [29–32]. We strongly caution against this development as much more basic science is required, while skirting this stage will likely lead to considerable waste of time and money. We wish to stress the need to develop better ways of assessing the likely dynamic nature of biased attention.

For the recently proposed new methods, further research of a more methodological nature would be required to overcome the sensitivity to variance that is not due to attention allocation, which we suggest is a major problem with these new methods. In the following paragraphs, we share a number of observations and thoughts and outline some key questions that we suggest will help the field to develop more appropriate methods to assess dynamics of attention allocation.

#### Characteristics of the DPT derived bias index

It is important to keep the DPT mechanics in mind when developing variability indices for DPT derived data. ABV is defined as ‘*SD*_(BI across bins)_ / mean RT_(CT+IT)_’. This formula is functionally related to the ‘coefficient of variation’ (*SD*/mean), which reflects variability in magnitude. Do note, however, that the ABV formula uses the mean RT rather than the mean BI over bins as the denominator. Importantly, while coefficients of variation allow comparisons of variability between measurements taken on different scales (for instance questionnaires that tap into the same construct), they are valid only for measures that can take only positive values. The reason is that a coefficient of variation, and also the ABV index, cannot discern between positive and negative values in the dataset that it is calculated on. The numerator (*SD*) is an absolute (i.e. always ‘positive’) value that reflects the ‘spread’ of observed values in either direction from the observed mean. The *SD* used in the ABV formula is the *SD* observed over a series of bias indices (one per bin). A bias index carries two separate ‘pieces’ of information: its absolute value (e.g. 40 vs. 20) indicates the magnitude of bias, while its sign (+20 vs. -20) indicates its direction. When calculating *SD* over a time series of bias indices, the information carried by the signs of the original values, the direction of bias, is lost. Moreover, any *SDs* value is relative to its associated mean value. Thus, when calculating ABV, the information on bias magnitude is lost as well. Something similar happens in the calculation of the TL-BS variability index. This index is conceptualized as the length of the TL-BS line (e.g. the line through a time series of bias indices). When we look at a TL-BS time series, the (actual) difference between any two consecutive TL-BS no longer reflects the magnitude of bias (i.e. its distance from bias = 0 is unknown) but is still meaningful in that its sign indicates the magnitude by which bias (assuming a sufficiently error-free measurement) is rising or falling. Yet by taking the absolute difference, this information gets ‘dropped’, and a relative increase in bias becomes numerically equal to a relative decrease in bias. As a result of these two steps (looking at relative rather than actual changes, and then taking the absolute value of these relative changes) the resulting index reflects neither bias direction nor magnitude. The TL-BS positive/negative indices do not suffer this problem to the same extent, as they reflect actual rather than relative values and thus retain information on the magnitude of bias, even though information on the direction is separated over the separate indices for positive and negative TL-BS. Here lies a major challenge for devising future new indices to analyze dynamics of biased attention based on DPT data. Because the sign of a bias index carries crucial information in addition to the information carried in the magnitude of the value, existing methods of indexing data variability will not readily translate.

A simple count of the times a time series of bias indices moving through the observed mean bias value may provide a simpler index of switching frequency, yet would also be sensitive to *SD* differences. Another alternative approach might be the use of MLM or SEM type approaches to try and tease apart variability of the RT from variability of the IT-CT difference (bias).

#### Redefining attention bias?

The notion that attention allocation is a dynamic process holds face validity and, although often implied, has never been forwarded so explicitly as in the pioneering papers proposing the ABV and TL-BS measures. Traditionally, the concept of attention allocation bias is often described using phrases such as ‘the tendency to attend more towards emotional/relevant stimuli’, which, if ‘more’ is taken to indicate ‘more often’, seems to reflect the intuitive notion that this is not an absolute phenomenon. On the other hand, the DPT literature is rife with rather absolute statements implying biases in certain directions to be generally observed among certain individuals. We would wager that most dot probe researchers recall exchanges in which, following an explanation of the mechanics of the DPT, the question was posed why so many trials are needed to assess this bias. Another familiar situation is individual participants asking researchers to inform them on the magnitude of their bias. The typical answer in the first situation is that bias is not an absolute phenomenon and that many trials are necessary to ‘catch’ a tendency to orient more often towards or away from emotional information among the many trials where this tendency does not prevail. The typical answer in the second situation is that bias does not necessarily manifest clearly in any individual participant, and may even only be reliably observed at a group level [24]. Critically, these answers are often followed by a statement indicating that the DPT typically exhibits high *SDs*. Such prototypical exchanges also raise the question of whether reaction time based tasks, such as the dot probe, are best suited for indexing attention allocation bias. Especially if we (re)define this bias more specifically as “expressed in fluctuating, phasic bursts, toward *or* away from target stimuli” ([1], p3), we should consider the possibility that RT based measures might lack the temporal resolution to index such fluctuations. This is partly due to the fact that response times are a composite of many sub processes, including attention orienting, response selection, motor initiation and execution, and the response processing by hard- and software. Among all these sub processes, we seek to distill the time required to execute just one, attention orientation, from the sum index of the recorded response time. This necessitates the use of large numbers of trials and samples. Thus, it is not surprising that tasks like the DPT typically yield high measurement error rates.

#### Overt versus covert orienting?

Another longstanding debate in this field that most researchers are aware of but that is not very often explicitly addressed in recent ABM and DPT publications is the question of overt versus covert orienting. Dot probe assessed bias is often regarded as a measure of covert attention orientation, i.e. a reallocation of attentional focus that is not necessarily accompanied by a physical reallocation in the form of a head-, or eye-movement, saccade or even micro-saccade. Especially for DPTs using very brief stimulus exposure times, covert attention orienting may be understood as a reorienting of attention that precedes and guides subsequent covert (physical) orienting [33]. In the past decade, eye-tracking technology has taken a high flight, yet the dot probe has remained the ‘gold standard’ because it may be capable of indexing something that eye-tracking can not: covert attention allocation. Paradoxically, for certain populations (most notably depressed patient and depression analogue samples), some agreement exists that biased attention orienting as measured by the DPT specifically occurs at longer stimulus durations (e.g. [34], yet see [35]) potentially reflecting a tendency to dwell on negative information more rather than automatic attention capturing [36,37]. Moreover, in the DPT based ABM literature it is most common to use a 500 ms stimulus duration, a time frame sufficiently long to suggest that this is not meant to assess initial, automatic, covert orienting of attention. In line with these developments, an increasing number of studies have utilized eye-tracking to assess overt biases, often comparing overt and DPT derived indices in the same population. A review of this literature is beyond the scope of the current paper (see for instance [38]), yet a renewed focus on the overt/covert question may help the field in redefining our concept of ‘attention allocation bias’ and what we should aim to measure. One particularly interesting recent study aimed at reducing overt eye-movements during a DPT assessment and found significant threat related BI specifically when analyzing trials in which no overt eye-movements were made, suggesting that DPT may indeed assess covert attention orienting even at longer (400 ms) stimulus durations [39].

#### Definition of dynamic?

Following the question of how to define attention allocation bias itself, the next question is how to define dynamics in attention allocation bias. Explanations provided in both the ABV and TL-BS papers seemingly combine two different definitions of ‘dynamic’, which we have tried to separately assess in the two dynamic bias simulations. On the one hand there is a notion of dynamic differences in the magnitude of bias. This notion leans heavily on the definition of bias implied in the DPT itself: a larger difference in RT between IT and CT trials reflects a larger magnitude of bias. However, in both the ABV and TL-BS papers attention variability appears to also be defined as a tendency to switch attention allocation towards and away from emotional information *more often*, i.e. at a higher frequency. We observe that our dynamic bias frequency simulation, in which an increasing frequency of switches was implemented, also resulted in differences in bias magnitude. The observed traditional BI within a bin in the ABV method is lower or higher as a result of the proportion of the trials belonging to a positive or negative bias subset. Similarly, the observed TL_BS calculated around the trials where bias is switching, yield a lower value than the TL-BS calculated for a trial pair were both trials belong to the same subset (i.e. both trials belonging to BI = +20 or -20 subset). Thus it may not be possible to completely tease apart dynamics as being due to increased frequency of switching or increased amplitude while switching, and likely even less so in actual observed data (see for instance Fig 1 in [1]) in which both frequency and amplitude seems to differ between the two datasets). Nonetheless, as we pursue better ways to index dynamics of attention allocation, it would be good to have a clear definition of what constitutes dynamics change and what the implications are of bias being dynamic in terms of either frequency or magnitude.

#### Validity of *SD*(BI) or *SD(RT)* as indices of psychopathology?

We believe that the sensitivity of the ABV and TL-BS indices to *SD*_(RT)_ in the absence of IT-CT differences renders these indicators problematic for empirical purposes. The DPT task (and similar other tasks) relies heavily on the notion that the presence of bias is to be inferred from a differential response between trials that differ on a critical feature. In the context of DPT this is whether the target appears on the location previously occupied by the emotion or the neutral stimulus. There exist no other systematic differences between DPT trials, including the fact that the participant is presented with both an emotional and a neutral stimulus on each trial. If the responses (RTs) for both trial types are numerically interchangeable (as they are in several of our simulations in which the underlying distributions for CT and IT trials were specified identically within each individual dataset *‘i’*), any observed statistical difference cannot be attributed to differential responses to different trial types, and therefore not be taken to indicate bias as meant to be indexed by the task.

Nonetheless, it is important to acknowledge that the recently proposed measures were found to be associated with arachnophobia status (Zvielli et al., study 1), daily smoking rate (Zvielli et al., study 2), and levels of PTSD and depression symptoms (Iacoviello et al., 2014). Many researchers in the field will recognize that analogue and patient groups tend to show larger SDs on both the mean RT for CT/IT trials, as well as bias indices, which often manifests as an undesired finding of heteroscedasticity (or ‘heterogeneity of variances’). On the other hand, Zvielli and colleagues found that mean RT differences between the groups were predictive of group status (study 1) and smoking rate (study 2), but understandably did not proceed to suggest that these are to be used in the future as functional indices of psychopathology related group differences in attention bias in the context of a DPT. The question thus becomes whether *SD* differences can be explained in a functional, theoretically relevant, way. A body of literature exists in which *SD* in reaction time tasks is thought to reflect ‘mental noise’, associating *SD*_(RT)_ differences observed in forced-choice tasks to measures associated with psychopathology such as neuroticism, cognitive control, and self-reported symptom levels [40]. This literature may have served as a starting point for Iacoviello and colleagues [2]. In the context of DPT data, it is important to recognize that mean RTs and bias indices each have their own distributions, which do not necessarily take the same form. I.e. RTs typically show a very skewed distribution, yet their resulting BIs much less so. Similarly, the *SD* for the RTs do not need to be directly related to the *SD* observed for bias. The currently presented results suggest that the ABV and TL-BS measures cannot discern *SD* at the RT level from *SD* at the bias level. A possible future research direction could be to develop better measures to assess the reliability of *SD* differences *for the bias index* between control and patient/analogue populations, without losing, as argued above, both vital pieces of information contained in a bias index, and in conjunction with a proper, falsifiable, theory on their cause.

#### Aim to reduce *SD*s?

A perhaps more recommendable approach would be to aim to improve the measurement of biased attention allocation itself by reducing *SD* at the RT level. As mentioned in the introduction, in recent years an increasing number of researchers have voiced concerns regarding the reliability and stability of measures such as the DPT. With the rising interest in DPT based potential new treatments (ABM) in mind, it seems more urgent than ever to focus on improving methodologies to establish reliable assessment of attention allocation bias.

#### Tasks other than DPT?

Our simulations are very specifically modeled after the dot probe task given that that is the task that the newly proposed indices were presented for. Yet, several principles demonstrated will likely also hold if similar methods are applied to other tasks that rely on a difference between two types of trials to demonstrate the presence or absence of a processing bias (difference). These include the emotional Stroop, single cueing, and emotional visual search tasks, for which it may similarly be argued that the bias meant to be indexed is likely a dynamic rather than a static phenomenon.

## Conclusion

Our simulation results show that in their current form, indices of bias variability derived from dot probe task data are unsuitable for empirical research purposes: for any significant difference observed on these indices it is impossible to know whether it reflects *SD* differences in the absence of bias or also a quality of bias dynamics. The authors proposing ABV and TL-BS indices have done the field a great service in demonstrating that new approaches are possible and clarifying our previously implicit understanding of attention allocation biases as being an inherently dynamic process. We sincerely hope that these efforts may serve as an impetus to the field to redefine our concepts and methods with the aim of increasing our understanding of, and ability to reliably measure, (components of) biased attention allocation.

## Supporting Information

S1 Fig Dot Probe Task–scope of application. (DOCX)

S1 Script ABV simulation script. (R)

S2 Script ABV simulation wrapper script. (R)

S3 Script TL-BS simulation script. (R)

S4 Script TL-BS simulation wrapper script. (R)

S5 Script Combined plot scripts. (R)

S1 Table ABV simulations—complete outcome tables. (DOCX)

S2 Table TL-BS simulations—complete outcome tables. (DOCX)
