# Supplementary material for: Capturing Dynamics of Biased Attention: Are New Attention Variability Measures the Way Forward?
Source: PLoS One. 2016 Nov 22;11(11):e0166600. doi: 10.1371/journal.pone.0166600 (PMC5119769; doi:10.1371/journal.pone.0166600)
Supplement: S1 Fig — (DOCX) [file pone.0166600.s001.docx]

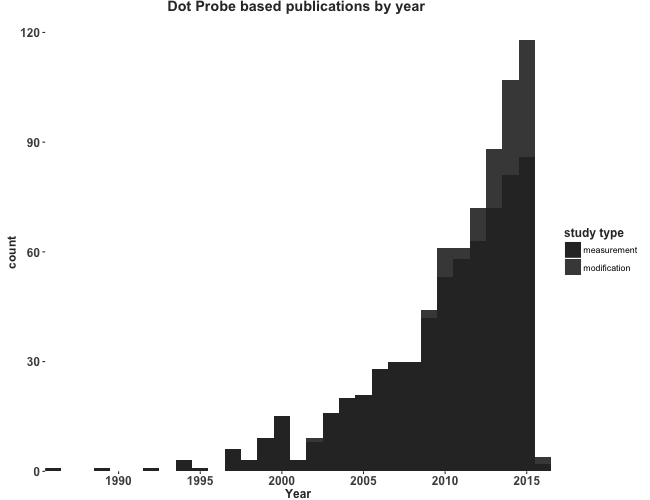


*S1 Figure 1. Dot Probe methodology based papers by year.*

*Based on a search in Elsevier www.scopus.com. Search string and processing details below.*

*S1 table 1: Dot Probe methodology based papers by domain and publication period*

| **Category** | **Publication period** | | | | | |  |
| --- | --- | --- | --- | --- | --- | --- | --- |
|  | [1986,1991] | (1991,1996] | (1996,2001] | (2001,2006] | (2006,2011] | (2011,2016] | *Total* |
| Sport_Psy | - | - | - | - | 1 | 2 | *3* |
| Military_Police_Professional_Safety | - | - | - | - | - | 4 | *4* |
| Reliability | - | - | - | 1 | 2 | 5 | *8* |
| Attachment_Theory | - | - | - | - | 2 | 6 | *8* |
| Aggression_and_Forensic | - | - | - | 3 | - | 7 | *10* |
| SelfEsteem_RejectionSensitivity | - | - | - | - | 3 | 11 | *14* |
| Language_Reading_Arithmethics | - | - | 2 | 1 | 1 | 4 | *8* |
| Aging_and_Eldery | - | - | - | 3 | 6 | 2 | *11* |
| Medical_Psy_nonSomatoform_incl_Cancer_Asthma_Pregnancy | - | - | 1 | - | 4 | 5 | *10* |
| Soc_Psy_Stereotyping_Racism | - | - | - | 1 | 5 | 7 | *13* |
| ActionPlanning_Goalmanagement | - | - | - | 1 | 3 | 8 | *12* |
| Treatment_Evaluation_Other | - | - | - | 1 | 3 | 10 | *14* |
| Evo_Psy_and_PrimateStudies | - | - | - | - | 7 | 8 | *15* |
| Genotyping_and_TwinStudies | - | - | - | - | 6 | 11 | *17* |
| Var_States_Political_Mortality_Humor_Power_Affiliation_etc | - | - | - | 2 | 9 | 5 | *16* |
| Soc_Psy_PersonalityFactors | - | - | 1 | 2 | 5 | 8 | *16* |
| Clin_Psy_Other | - | - | - | 4 | 4 | 9 | *17* |
| Neuroendocrinology | - | - | - | 1 | 5 | 14 | *20* |
| Visual_and_CrossModality_processing | - | - | 5 | 4 | 8 | 12 | *29* |
| Clin_Psy_Stress_and_Somatoform | - | - | 1 | 5 | 14 | 17 | *37* |
| EEG_Imaging_Psychophys | - | - | 2 | 4 | 21 | 36 | *63* |
| Clin_Psy_Impulsivity_SubstanceAbuse_Food | - | - | 3 | 19 | 41 | 64 | *127* |
| Clin_Psy_Mood_Disorders | 2 | 5 | 21 | 42 | 76 | 134 | *280* |
| *Total* | *2* | *5* | *36* | *94* | *226* | *389* | *752* |

**Search string & post-processing methods:**

Search string: ( (TITLE-ABS-KEY (( "dot probe" OR "probe detection task" OR "visual probe" OR "attentional probe task" OR "probe classification task" OR "atten* bias modification" ) OR ( "atten* retrain*" AND "probe" )) AND TITLE-ABS-KEY ( "bias*" OR "atten*" )) AND NOT TITLE ( "sys* review" OR "meta anal*" ) ) AND DOCTYPE (ar OR ip) AND LANGUAGE ("English" ) AND SRCTYPE ( "j" )

A scopus search executed on 09-12-2015 results in 791 records.

Records were downloaded and processed using the following rules:

- manual check & removal of records published before 1986
  - 6 records removed
- manual check & removal of records containing keywords ("quantum", "ferromagnetic", "D-dot", "B-dot") in search fields Abstract, Index.Keywords & Author.Keywords as these refer to a method in quantum physics.
  - 6 records removed
- manual check for records containing keywords ("chem") in search fields Abstract, Index.Keywords & Author.Keywords - 14 records with keyword chem each do report on dot probe tasks and are retained
- Manual evaluation of Titles & Abstracts for records published in journals that do NOT contatin the following words in the journal title: “"psych", "behav", "cogni", "disord", "neuro", "clinic", "emotion", "alcohol", "tobacco", "addiction", "pain", "anxiety", "treatment", "schizoph", "stress", "appetite", "personality"
  - 8 records removed
- remove duplicate records based on a search for identical Abstracts
  - 2 records removed
- manual check & removal of records containing keywords ("secondary task", "concurrent-task", “dual task”, “preview search”, “multiple object tracking” or “IOR” in search fields Abstract, Index.Keywords & Author.Keywords as these refer to other dot based methods.
  - 12 records removed
- tagging of bias modification studies based on presence of the following keywords in Abstract, Author.Keywords, and Index.Keywords: “bias modific*", "atten* retrain*", "bias-modification", "disengagement training", "induction of attentional", "encouraged to attend to either affective"
- Categorisation of records based on keyword searches – detailed separately below.
- Categorisation keywords were added based on manual evaluation of non-categorised records.
- Removal of Titles & Abstracts that remain uncategorised.
  - 7 non-dot-probe and 1 non-empirical records removed
- A total of 752 records was categorised and retained in the final dataset.

**Record categorisation:**

The table below lists the keywords used for each category. For representation in the figure and table above, records that showed up in more than one category were assigned to the smallest category. This was achieved by ordering the string searches based on the resulting numbers. In the tables the categories are represented in the order in which the categorization search strings were executed, with each paper being assigned to the first category which’ keywords appear in the paper’s abstract or keywords. The search strings used were of the form:

dataframe$category <- as.numeric(grepl(paste(c("(?i) keyword1", "keyword2", "keyword3"), collapse = "|"), paste(dataframe$Abstract, dataframe$Index.Keywords, dataframe$Author.Keywords)))

Writing the categorization part of the script has been a recursive process in which keywords were added based on manual evaluation of non-categorised records. Nonetheless we did aim for as much an automated process as possible and this categorization should be regarded as somewhat crude.

| **Category** | **keywords** |
| --- | --- |
| Sport_Psy | "(?i) sport", "tennis", "exercise-related images"), collapse = "\|") |
| Military_Police_Professional_Safety | "(?i) military", "police", "workplace safety"), collapse = "\|") |
| Reliability | "(?i)retest reliability"), collapse = "\|") |
| Attachment_Theory | "(?i) attachment theory", "attachment-related", "Anxiously-attached", "attachment style"), collapse = "\|") |
| Aggression_and_Forensic | "(?i) forensic", "delinq", "psychopathy", "proactive aggression", "guilty", "crime", "criminal", "recidivis*", "offenders"), collapse = "\|") |
| SelfEsteem_RejectionSensitivity | "(?i) self-esteem", "rejection sensitivity", "appearance concern", "social exclusion", "upward social comparison"), collapse = "\|") |
| Language_Reading_Arithmethics | "(?i) chinese words", "Word Recognition", "word-reading", "spatial-numerical", "letter memory", "arithmetical"), collapse = "\|") |
| Aging_and_Eldery | ("(?i) aging-related", "age-related", "older cohort", "older adults"), collapse = "\|") |
| Medical_Psy_nonSomatoform_incl_Cancer_Asthma_Pregnancy | "(?i) cancer", "post-operative"," migraine", "Williams syndrome", "pulmonary disease", "asthma", "pregnancy"), collapse = "\|") |
| Soc_Psy_Stereotyping_Racism | "(?i) stereotype","outgroup", "out-group", "moral value", "Other-Race", "famous persons", "famous faces", "fitness and fatness"), collapse = "\|") |
| ActionPlanning_Goalmanagement | "(?i) voluntary attention", "current goal", "motivational relevance", "motivational aspects", "motivational states", "planning future actions", "Inhibition-Induced Forgetting"), collapse = "\|") |
| Treatment_Evaluation_Other | "(?i) citalopram", "D3 receptor antagonist GSK598809", "acupuncture", "High-density negative ion", "fluoxetin", "escitalopram", "tDCS", "Attention Process Training-II"), collapse = "\|") |
| Evo_Psy_and_PrimateStudies | "(?i)altruistic", "progeny survival", "direct gaze", "sexual motivation", "attractiveness", "primates", "monkey*", "evolutio*"), collapse = "\|") |
| Genotyping_and_TwinStudies | "(?i) genetic", "genotype", "monozygotic", "DBH", "MAOA", "HTTLPR"), collapse = "\|") |
| Var_States_Political_Mortality_Humor_Power_Affiliation_etc | "(?i) political ideology", "reminders of mortality on", "humor", "envy", "body dissatisfaction", "height dissatisfaction", "urban settings", "attention capture by sexual stimuli", "power and affiliation", "power vs. affiliation", "customer blogs"), collapse = "\|") |
| Soc_Psy_PersonalityFactors | "(?i)personality", "neuroticism", "MMPI-2 Type A"), collapse = "\|")  Clin_Psy_Other "(?i) schizophrenia", "autism", "delusion", "insomnia", "visual neglect", "sexual dysfunction", "Narcissistic personality disorder","narcissists", "Compulsive sexual behaviour", "body dysmorphic disorder"), collapse = "\|") |
| Neuroendocrinology | "(?i) noradrenergic", "hormone", "pheromone", "testoster", "cortisol", "oxytoc", "hydrocortisone"), collapse = "\|") |
| Visual_and_CrossModality_processing <- | as.numeric(grepl(paste(c("(?i) different orientation singletons", "crossmodal", "bias towards hands", "relevance orienting", "guidance of selective", "grasp cueing", "cross-modal", "saccade preparation", "Looming Motion", "object recognition ", "far-hand", "inhibition of return", "Luminance Detection", "auditory attention", "motion extrapolation", "spatial uncertainty", "sensory input", "associative learning", "visual input", "color-in-context theory", "auditory emotional information"), collapse = "\|") |
| Clin_Psy_Stress_and_Somatoform | "(?i) IBD", "bowel", "chronic pain", "pain-related attention", "functional abdominal pain", "high blood pressure", "Idiopathic environmental intolerance", "pain catastrophizing", "level of pain"), collapse = "\|") |
| EEG_Imaging_Psychophys | "(?i)EEG", "Evoked Potentials", "(?-i)ERP", "(?i)MRI", "Magnetic Resonance Imaging", "PET", "source localization", "respiratory sinus arrhythmia"), collapse = "\|") |
| Clin_Psy_Impulsivity_SubstanceAbuse_Food | "(?i) substance abuse", "smoking", "craving", "food cue", "obese", "satiety", "chocolate", "calorific", "hunger", "dieters","alcohol", "cocaine", "ketamine", "eating disorder", "OCD", "ADHD", "AD/HD", "addict*", "nicotine", "d-amphetamine", "cigarette"), collapse = "\|") |
| Clin_Psy_Mood_Disorders | "(?i) anx", "depr", "dysphor", "phobi", "fobi*", "fear", "panic", "threat", "worry", "negative affect", "PTSD", "posttraumatic stress", "intrusive memories", "bipolar", "aversive emotional states", "emotion-antecedent", "negative attentional bias", "trait rumination", "relevance of negative", "positive affectivity", "emotional vulnerability", " affective symptoms"), collapse = "\|") |
