# Supplementary material for: Capturing Dynamics of Biased Attention: Are New Attention Variability Measures the Way Forward?
Source: PLoS One. 2016 Nov 22;11(11):e0166600. doi: 10.1371/journal.pone.0166600 (PMC5119769; doi:10.1371/journal.pone.0166600)
Supplement: S1 Table — (DOCX) [file pone.0166600.s007.docx]

| **SD increasing**  ***2 points/run: 30 - 48*** | | | | | | | | | | | |
| --- | --- | --- | --- | --- | --- | --- | --- | --- | --- | --- | --- |
|  | **set SD change groups** | **ABV** | | | | | **Bias Index** | | | | |
|  |  | **change groups** | | **control groups** | | **% sig. t-tests** | **change groups** | | **control groups** | | **% sig. t-tests** |
| **run** |  | **Mean** | **95% CI** | **Mean** | **95% CI** |  | **Mean** | **95% CI** | **Mean** | **95% CI** |  |
| **1** | ***30*** | .024 | [.024, .024] | .024 | [.024, .024] | 5.9 | .0 | [-.1, .1] | -.0 | [-.1, .0] | 6.2 |
| **2** | ***32*** | .026 | [.025, .026] | .024 | [.024, .024] | 13.5 | .0 | [-.1, .1] | -.0 | [-.1, .1] | 5.6 |
| **3** | ***34*** | .027 | [.027, .027] | .024 | [.024, .024] | 34.6 | -.1 | [-.1, .0] | -.0 | [-.1, .1] | 4.8 |
| **4** | ***36*** | .029 | [.029, .029] | .024 | [.024, .024] | 63.6 | -.1 | [-.1, .0] | -.0 | [-.1, .1] | 4.7 |
| **5** | ***38*** | .030 | [.030, .030] | .024 | [.024, .024] | 83.7 | .0 | [-.1, .1] | .1 | [.0, .1] | 5.2 |
| **6** | ***40*** | .032 | [.032, .032] | .024 | [.024, .024] | 96.0 | .0 | [-.1, .0] | -.0 | [-.1, .0] | 4.3 |
| **7** | ***42*** | .033 | [.033, .034] | .024 | [.024, .024] | 98.0 | -.1 | [-.2, .0] | -.0 | [-.1, .1] | 5.4 |
| **8** | ***44*** | .035 | [.035, .035] | .024 | [.024, .024] | 99.8 | -.1 | [-.2, .1] | -.0 | [-.1, .0] | 5.6 |
| **9** | ***46*** | .037 | [.036, .037] | .024 | [.024, .024] | 100 | .1 | [-.1, .2] | -.0 | [-.1, .1] | 5.2 |
| **10** | ***48*** | .038 | [.038, .038] | .024 | [.024, .024] | 100 | .1 | [-.1, .2] | .0 | [-.0, .1] | 4.8 |
| *Results for the ABV SD rt increasing simulation, consisting of ten runs r of 1000 ‘studies’ j, each with 52 ‘individuals’ i. ‘Set SD change groups’ is the SD value used for generating rt values for the change groups. ‘% sig. t-tests’ is the percentage of studies in which t-tests for group differences return p < .05.*  *SD at the rt level is kept at 30 for control groups. Mean rt is kept at 600 for both groups and both trial types, no bias is created* | | | | | | | | | | | |

| **Mean increasing**  ***20 points/run: 600 - 780*** | | | | | | | | | | | |
| --- | --- | --- | --- | --- | --- | --- | --- | --- | --- | --- | --- |
|  | **set mean change groups** | **ABV** | | | | | **Bias Index** | | | | |
|  |  | **change groups** | | **control groups** | | **% sig. t-tests** | **change groups** | | **control groups** | | **% sig. t-tests** |
| **run** |  | **Mean** | **95% CI** | **Mean** | **95% CI** |  | **Mean** | **95% CI** | **Mean** | **95% CI** |  |
| **1** | ***600*** | .024 | [.024, .024] | .024 | [.024, .024] | 4.9 | .0 | [.0, .1] | .0 | [-.1, .1] | 5.6 |
| **2** | ***620*** | .023 | [.023, .023] | .024 | [.024, .024] | 7.1 | -.1 | [-.1, .0] | .0 | [-.0, .1] | 4.8 |
| **3** | ***640*** | .022 | [.022, .022] | .024 | [.024, .024] | 13.9 | .0 | [-.1, .1] | .0 | [-.1, .1] | 4.9 |
| **4** | ***660*** | .022 | [.022, .022] | .024 | [.024, .024] | 23.4 | .0 | [-.1, .1] | .0 | [-.1, .1] | 4.9 |
| **5** | ***680*** | .021 | [.021, .021] | .024 | [.024, .024] | 32.2 | -.1 | [-.1, .0] | .0 | [.0, .1] | 5.5 |
| **6** | ***700*** | .021 | [.020, .021] | .024 | [.024, .024] | 46.4 | .0 | [-.1, .1] | .0 | [-.1, .0] | 5.5 |
| **7** | ***720*** | .020 | [.020, .020] | .024 | [.024, .024] | 61.9 | .0 | [-.1, .0] | .0 | [-.1, .1] | 4.5 |
| **8** | ***740*** | .019 | [.019, .019] | .024 | [.024, .024] | 76.0 | .1 | [.0, .1] | -.1 | [-.1, .0] | 5.3 |
| **9** | ***760*** | .019 | [.019, .019] | .024 | [.024, .024] | 84.0 | .1 | [.0, .1] | .0 | [-.1, .1] | 4.1 |
| **10** | ***780*** | .018 | [.018, .018] | .024 | [.024, .024] | 91.1 | .0 | [-.1, .0] | .0 | [.0, .1] | 5.2 |
| *Results for the ABV Mean rt increasing simulation, consisting of ten runs r of 1000 ‘studies’ j, each with 52 ‘individuals’ i. ‘Set mean change groups’ is the mean value used for generating rt values for the change groups. ‘% sig. t-tests’ is the percentage of studies in which t-tests for group differences return a p < .05.*  *Mean rt for control groups is kept at 600. SD at the rt level is kept at 30 for both groups and both trial types. No bias is created.* | | | | | | | | | | | |

| **Bias increasing**  ***3 points/run: 0 - 27*** | | | | | | | | | | | |
| --- | --- | --- | --- | --- | --- | --- | --- | --- | --- | --- | --- |
|  | **set bias change groups** | **ABV** | | | | | **Bias Index** | | | | |
|  |  | **change groups** | | **control groups** | | **% sig. t-tests** | **change groups** | | **control groups** | | **% sig. t-tests** |
| **run** |  | **Mean** | **95% CI** | **Mean** | **95% CI** |  | **Mean** | **95% CI** | **Mean** | **95% CI** |  |
| **1** | **0** | .024 | [.024, .024] | .024 | [.024, .024] | 4.5 | -.0 | [-.10, .03] | .0 | [-.1, .0] | 4.3 |
| **2** | **3** | .024 | [.024, .024] | .024 | [.024, .024] | 4.4 | 3.1 | [2.99, 3.12] | .0 | [-.1, .1] | 49.7 |
| **3** | **6** | .024 | [.024, .024] | .024 | [.024, .024] | 4.7 | 6.0 | [5.95, 6.09] | .0 | [-.1, .1] | 96.7 |
| **4** | **9** | .024 | [.024, .024] | .024 | [.024, .024] | 3.8 | 9.0 | [8.91, 9.05] | .0 | [.0, .1] | 100 |
| **5** | **12** | .024 | [.024, .024] | .024 | [.024, .024] | 5.3 | 12.0 | [11.97, 12.10] | .0 | [-.1, .1] | 100 |
| **6** | **15** | .024 | [.024, .024] | .024 | [.024, .024] | 3.7 | 15.0 | [14.92, 15.05] | -.1 | [-.1, .0] | 100 |
| **7** | **18** | .024 | [.024, .024] | .024 | [.024, .024] | 4.2 | 18.0 | [17.93, 18.06] | .0 | [-.1, .1] | 100 |
| **8** | **21** | .024 | [.024, .024] | .024 | [.024, .024] | 5.1 | 21.0 | [20.94, 21.07] | .0 | [-.1, .1] | 100 |
| **9** | **24** | .024 | [.024, .024] | .024 | [.024, .024] | 5.9 | 23.9 | [23.86, 24.00] | .0 | [-.1, .1] | 100 |
| **10** | **27** | .024 | [.024, .024] | .024 | [.024, .024] | 6.1 | 27.1 | [27.02, 27.15] | -.1 | [-.1, .0] | 100 |
| *Results for the ABV Bias increasing simulation, consisting of ten runs r of 1000 ‘studies’ j, each with 52 ‘individuals’ i.‘Set bias change groups’ is the mean rt difference between incongruent and congruent trials implied when generating rt values for the change groups.*  *‘% sig. t-tests’ is the percentage of studies in which t-tests for group differences return a p < .05.*  *Mean rt were kept at 600 for control groups. SD at the rt level is kept at 30 for both groups and both trial types.* | | | | | | | | | | | |

| **Dynamic bias frequency increasing**  ***bias = +\|- 20, switches: 0 - 9*** | | | | | | | | | | | |
| --- | --- | --- | --- | --- | --- | --- | --- | --- | --- | --- | --- |
|  | **n bias switch** | **ABV** | | | | | **Bias Index** | | | | |
|  |  | **change groups** | | **control groups** | | **% sig. t-tests** | **change groups** | | **control groups** | | **% sig. t-tests** |
| **run** |  | **Mean** | **95% CI** | **Mean** | **95% CI** |  | **Mean** | **95% CI** | **Mean** | **95% CI** |  |
| **1** | ***0*** | .024 | [.024, .024] | .024 | [.024, .024] | 4.5 | 20.1 | [20.0, 20.1] | 20.0 | [20.0, 20.1] | 5.0 |
| **2** | ***1*** | .040 | [.040, .040] | .024 | [.024, .024] | 100 | 0.0 | [-.1, .1] | 20.0 | [19.9, 20.1] | 100 |
| **3** | ***2*** | .035 | [.035, .035] | .024 | [.024, .024] | 100 | 6.8 | [6.8, 6.9] | 20.0 | [19.9, 20.1] | 100 |
| **4** | ***3*** | .040 | [.040, .040] | .024 | [.024, .024] | 100 | .0 | [.0, .1] | 20.0 | [19.9, 20.1] | 100 |
| **5** | ***4*** | .034 | [.034, .034] | .024 | [.024, .024] | 99.3 | 4.1 | [4.0, 4.1] | 20.0 | [19.9, 20.1] | 100 |
| **6** | ***5*** | .033 | [.033, .033] | .024 | [.024, .024] | 99.5 | -.5 | [-.6, -.4] | 19.9 | [19.9, 20.0] | 100 |
| **7** | ***6*** | .031 | [.031, .031] | .024 | [.024, .024] | 91.9 | 3.6 | [3.5, 3.7] | 20.0 | [19.9, 20.0] | 100 |
| **8** | ***7*** | .040 | [.040, .040] | .024 | [.024, .024] | 100 | 0.0 | [-.1, .1] | 20.0 | [20.0, 20.1] | 100 |
| **9** | ***8*** | .030 | [.030, .030] | .024 | [.024, .024] | 87.8 | 3.1 | [3.0, 3.1] | 20.0 | [20.0 20.1] | 100 |
| **10** | ***9*** | .028 | [.028, .028] | .024 | [.024, .024] | 54.4 | 0.0 | [-.1, .1] | 20.0 | [20.0, 20.1] | 100 |
| *Results for the ABV dynamic increasing simulation, consisting of ten runs r of 1000 ‘studies’ j, each with 52 ‘individuals’ i.‘n bias switch’ is the number of times bias switches from +20 to -20 and vice versa, which happens after every 1/r trials. ‘% sig. t-tests’ is the percentage of studies in which t-tests for group differences return a p < .05.*  *Bias is kept at +20 for control groups. SD at the rt level is kept at 30 for both groups and both trial types.* | | | | | | | | | | | |

| **Dynamic bias magnitude increasing**  ***Bias sign switches = 3, bias magnitude = +\|- 20 - 46*** | | | | | | | | | | | |
| --- | --- | --- | --- | --- | --- | --- | --- | --- | --- | --- | --- |
|  | **Set bias** | **ABV** | | | | | **Bias Index** | | | | |
|  |  | **change groups** | | **control groups** | | **% sig. t-tests** | **change groups** | | **control groups** | | **% sig. t-tests** |
| **run** |  | **Mean** | **95% CI** | **Mean** | **95% CI** |  | **Mean** | **95% CI** | **Mean** | **95% CI** |  |
| **1** | ***20*** | .040 | [.040, .040] | .040 | [.040, .040] | 6.1 | 0.0 | [-0.1, .08] | -0.0 | [-0.1, .1] | 4.8 |
| **2** | ***23*** | .044 | [.044, .044] | .040 | [.040, .040] | 37.5 | -0.0 | [-0.1, .04] | 0.0 | [-0.1, .1] | 4.4 |
| **3** | ***26*** | .048 | [.048, .048] | .040 | [.040, .040] | 90.8 | 0.0 | [-0.1, .09] | -0.0 | [-0.1, .1] | 3.9 |
| **4** | ***29*** | .053 | [.053, .053] | .040 | [.040, .040] | 99.8 | -0.1 | [-0.1, .02] | 0.0 | [-0.0, .1] | 4.8 |
| **5** | ***32*** | .058 | [.057, .058] | .040 | [.040, .040] | 100 | -0.0 | [-0.1, .07] | -0.0 | [-0.1, .1] | 4.0 |
| **6** | ***35*** | .062 | [.062, .062] | .040 | [.040, .040] | 100 | 0.0 | [-0.0, .10] | 0.1 | [.0, .2] | 4.6 |
| **7** | ***38*** | .067 | [.067, .067] | .040 | [.040, .040] | 100 | 0.0 | [-0.1, .08] | -0.0 | [-0.1, .1] | 6.2 |
| **8** | ***41*** | .072 | [.072, .072] | .040 | [.040, .040] | 100 | -0.1 | [-0.1, .01] | -0.0 | [-0.1, .1] | 5.3 |
| **9** | ***44*** | .077 | [.077, .078] | .040 | [.040, .040] | 100 | 0.0 | [-0.1, .08] | -0.1 | [-0.1, -0.0] | 5.1 |
| **10** | ***47*** | .083 | [.082, .083] | .040 | [.040, .040] | 100 | -0.0 | [-0.1, .04] | 0.0 | [-0.0, .1] | 3.7 |
| *Results for the ABV dynamic bias magnitude increasing simulation, consisting of ten runs r of 1000 ‘studies’ j, each with 52 ‘individuals’ i.. Set bias is the magnitude of bias in the change groups. Bias switches sign (+20 to-20 and v.v.) three times in all datasets (change and control groups). ‘% sig. t-tests’ is the percentage of studies in which t-tests for group differences return a p < .05.*  *Bias magnitude is kept at +\|- 20 for control groups. SD at the rt level is kept at 30 for both groups and both trial types.* | | | | | | | | | | | |
