# Supplementary material for: Capturing Dynamics of Biased Attention: Are New Attention Variability Measures the Way Forward?
Source: PLoS One. 2016 Nov 22;11(11):e0166600. doi: 10.1371/journal.pone.0166600 (PMC5119769; doi:10.1371/journal.pone.0166600)
Supplement: S2 Table — (DOCX) [file pone.0166600.s008.docx]

| **SD increasing**  ***2 points/run: 30 - 48*** | | | | | | | | | | | |
| --- | --- | --- | --- | --- | --- | --- | --- | --- | --- | --- | --- |
|  | **set SD change groups** | **TL-BS variability** | | | | | **Bias Index** | | | | |
|  |  | **change groups** | | **control groups** | | **% sig. t-tests** | **change groups** | | **control groups** | | **% sig. t-tests** |
| **run** |  | **Mean** | **95% CI** | **Mean** | **95% CI** |  | **Mean** | **95% CI** | **Mean** | **95% CI** |  |
| **1** | ***30*** | 28.1 | [28.0, 28.1] | 28.1 | [28.0, 28.1] | 4.2 | 0.0 | [-0.1, 0.1] | 0.0 | [-0.1, 0.1] | 4.5 |
| **2** | ***32*** | 29.9 | [29.8, 30.0] | 28.0 | [28.0, 28.1] | 27.8 | 0.1 | [-0.1, 0.2] | 0.1 | [0.0, 0.2] | 5.3 |
| **3** | ***34*** | 31.7 | [31.7, 31.8] | 28.1 | [28.0, 28.1] | 72.8 | -0.1 | [-0.2, 0.1] | 0.0 | [-0.1, 0.1] | 4.4 |
| **4** | ***36*** | 33.5 | [33.4, 33.5] | 28.0 | [28.0, 28.1] | 96.2 | 0.0 | [-0.1, 0.2] | 0.1 | [0.0, 0.2] | 5.4 |
| **5** | ***38*** | 35.5 | [35.7, 35.5] | 28.0 | [28.0, 28.1] | 100 | 0.0 | [-0.1, 0.2] | 0.1 | [-0.1, 0.2] | 4.7 |
| **6** | ***40*** | 37.3 | [37.2, 37.3] | 28.1 | [28.0, 28.1] | 100 | 0.0 | [-0.2, 0.1] | 0.2 | [0.0, 0.3] | 5.2 |
| **7** | ***42*** | 39.1 | [39,0 39.1] | 28.1 | [28.0, 28.1] | 100 | 0.0 | [-0.2, 0.2] | 0.0 | [-0.1, 0.1] | 5.3 |
| **8** | ***44*** | 40.9 | [40.8, 41.0] | 28.0 | [28.0, 28.1] | 100 | 0.1 | [-0.1, 0.2] | 0.1 | [-0.1, 0.2] | 5.1 |
| **9** | ***46*** | 42.7 | [42.6, 42.8] | 28.0 | [28.0, 28.1] | 100 | -0.1 | [-0.3, 0.1] | 0.0 | [-0.1, 0.1] | 4.9 |
| **10** | ***48*** | 44.6 | [44.5, 44.7] | 28.0 | [28.0, 28.1] | 100 | 0.0 | [-0.2, 0.2] | 0.0 | [-0.1, 0.2] | 5.1 |
|  | **set SD change groups** | **average TL-BS positive** | | | | | **peak TL-BS positive** | | | | |
|  |  | **change groups** | | **control groups** | | **% sig. t-tests** | **change groups** | | **control groups** | | **% sig. t-tests** |
| **run** |  | **Mean** | **95% CI** | **Mean** | **95% CI** |  | **Mean** | **95% CI** | **Mean** | **95% CI** |  |
| **1** | ***30*** | 33.5 | [33.4, 33.6] | 33.4 | [33.3, 33.5] | 4.6 | 82.5 | [82.3, 82.8] | 82.5 | [82.3, 82.8] | 6.2 |
| **2** | ***32*** | 35.7 | [35.6 35.8] | 33.5 | [33.4, 33.6] | 13.4 | 87.9 | [87.6, 88.2] | 82.8 | [82.5, 83.0] | 11.9 |
| **3** | ***34*** | 37.9 | [37.8, 38.0] | 33.5 | [33.4, 33.6] | 42.1 | 93.6 | [93.3, 93.9] | 82.5 | [82.2, 82.8] | 37.5 |
| **4** | ***36*** | 40.0 | [39.9, 40.1] | 33.4 | [33.3, 33.5] | 73.3 | 98.6 | [98.2, 98.9] | 82.6 | [82.3, 82.9] | 63.3 |
| **5** | ***38*** | 42.3 | [42.2, 42.5] | 33.4 | [33.3, 33.5] | 92.0 | 104.6 | [104.3, 105.0] | 82.6 | [82.3, 82.9] | 87.3 |
| **6** | ***40*** | 44.5 | [44.3, 44.6] | 33.5 | [33.4, 33.6] | 98.2 | 109.7 | [109.4, 110.1] | 82.8 | [82.6, 83.1] | 96.5 |
| **7** | ***42*** | 46.7 | [46.6, 46.9] | 33.4 | [33.3, 33.5] | 99.6 | 115.6 | [115.2, 116.0] | 82.6 | [82.3, 82.9] | 99.2 |
| **8** | ***44*** | 49.0 | [48.8, 49.1] | 33.4 | [33.3, 33.5] | 99.9 | 120.9 | [120.5, 121.3] | 82.8 | [82.5, 83.0] | 99.7 |
| **9** | ***46*** | 51.1 | [50.9, 51.2] | 33.4 | [33.3, 33.5] | 100 | 126.3 | [125.9, 126.7] | 82.6 | [82.4, 82.9] | 100 |
| **10** | ***48*** | 53.5 | [53.4, 53.7] | 33.3 | [33.2, 33.4] | 100 | 132.2 | [131.8, 132.6] | 82.4 | [82.1, 82.6] | 100 |
|  | **set SD change groups** | **average TL-BS negative** | | | | | **peak TL-BS negative** | | | | |
|  |  | **change groups** | | **control groups** | | **% sig. t-tests** | **change groups** | | **control groups** | | **% sig. t-tests** |
| **run** |  | **Mean** | **95% CI** | **Mean** | **95% CI** |  | **Mean** | **95% CI** | **Mean** | **95% CI** |  |
| **1** | ***30*** | -33.3 | [-33.4, -33.2] | -33.5 | [-33.6, -33.4] | 4.3 | -82.5 | [-82.8, -82.3] | -82.6 | [-82.9, -82.3] | 4.8 |
| **2** | ***32*** | -35.6 | [-35.7, -35.5] | -33.3 | [-33.4, -33.2] | 15.0 | -87.8 | [-88.1, -87.5] | -82.3 | [-82.6, -82.0] | 13.3 |
| **3** | ***34*** | -37.9 | [-38.1, -37.8] | -33.5 | [-33.6, -33.4] | 43.1 | -93.5 | [-93.8, -93.2] | -82.4 | [-82.7, -82.2] | 37.8 |
| **4** | ***36*** | -40.0 | [-40.2, -39.9] | -33.3 | [-33.4, -33.2] | 75.5 | -98.9 | [-99.2, -98.6] | -82.3 | [-82.6, -82.0] | 66.8 |
| **5** | ***38*** | -42.4 | [-42.6, -42.3] | -33.4 | [-33.5, -33.3] | 93.1 | -104.6 | [-105.0, -104.3] | -82.4 | [-82.7, -82.1] | 87.9 |
| **6** | ***40*** | -44.6 | [-44.7, -44.5] | -33.4 | [-33.5, -33.3] | 98.4 | -110.0 | [-110.3, -109.6] | -82.4 | [-82.7, -82.1] | 96.6 |
| **7** | ***42*** | -46.7 | [-46.9, -46.6] | -33.4 | [-33.5, -33.3] | 99.9 | -115.5 | [-115.9, -115.1] | -82.4 | [-82.7, -82.1] | 99.4 |
| **8** | ***44*** | -48.9 | [-49.1, -48.8] | -33.4 | [-33.5, -33.3] | 100 | -120.8 | [-121.2, -120.4] | -82.4 | [-82.6, -82.1] | 99.9 |
| **9** | ***46*** | -51.2 | [-51.4, -51.1] | -33.4 | [-33.5, -33.3] | 100 | -126.6 | [-127.0, -126.2] | -82.6 | [-82.8, -82.3] | 100 |
| **10** | ***48*** | -53.5 | [-53.7, -53.3] | -33.4 | [-33.5, -33.3] | 100 | -132.1 | [-132.5, -131.7] | -82.5 | [-82.8, -82.2] | 100 |
| *Results for the TL-BS SD rt increasing simulation, consisting of ten runs r of 1000 ‘studies’ j, each with 52 ‘individuals’ i. ‘Set SD change groups’ is the SD value used for generating rt values for the change groups. ‘% sig. t-tests’ is the percentage of studies in which t-tests for group differences return p < .05.*  *SD at the rt level is kept at 30 for control groups. Mean rt is kept at 600 for both group types. No bias is created.* | | | | | | | | | | | |

| **Mean increasing**  ***20 points/run: 600 - 780*** | | | | | | | | | | | |
| --- | --- | --- | --- | --- | --- | --- | --- | --- | --- | --- | --- |
|  | **set mean change groups** | **TL-BS variability** | | | | | **Bias Index** | | | | |
|  |  | **change groups** | | **control groups** | | **% sig. t-tests** | **change groups** | | **control groups** | | **% sig. t-tests** |
| **run** |  | **Mean** | **95% CI** | **Mean** | **95% CI** |  | **Mean** | **95% CI** | **Mean** | **95% CI** |  |
| **1** | ***600*** | 28.1 | [28.0, 28.1] | 28.1 | [28.0, 28.1] | 4.8 | 0.1 | [-0.1, 0.2] | -0.1 | [-0.2, 0.1] | 5.0 |
| **2** | ***620*** | 28.0 | [27.9, 28.1] | 28.1 | [28.0, 28.1] | 5.4 | 0.0 | [-0.1, 0.1] | -0.1 | [-0.2, 0.0] | 4.8 |
| **3** | ***640*** | 28.0 | [27.9, 28.0] | 28.0 | [28.0, 28.1] | 4.6 | 0.0 | [-0.1, 0.1] | 0.0 | [-0.1, 0.1] | 5.3 |
| **4** | ***660*** | 28.1 | [28.0, 28.2] | 28.0 | [28.0, 28.1] | 4.3 | 0.0 | [-0.1, 0.1] | 0.0 | [-0.2, 0.1] | 3.8 |
| **5** | ***680*** | 28.0 | [28.0, 28.1] | 28.0 | [28.0, 28.1] | 4.4 | -0.1 | [-0.2, 0.1] | 0.0 | [-0.1, 0.1] | 3.7 |
| **6** | ***700*** | 28.0 | [27.9, 28.0] | 28.1 | [28.0, 28.1] | 4.7 | -0.1 | [-0.2, 0.1] | -0.1 | [-0.2, 0.1] | 3.7 |
| **7** | ***720*** | 28.0 | [28.0, 28.1] | 28.0 | [28.0, 28.1] | 5.0 | 0.0 | [-0.2, 0.1] | 0.1 | [-0.0, 0.2] | 5.1 |
| **8** | ***740*** | 28.1 | [28.0, 28.1] | 28.0 | [28.0, 28.1] | 6.0 | 0.0 | [-0.1, 0.1] | 0.0 | [-0.1, 0.1] | 5.3 |
| **9** | ***760*** | 28.0 | [28.0, 28.1] | 28.1 | [28.0, 28.1] | 5.1 | 0.0 | [-0.1, 0.1] | 0.0 | [-0.1, 0.1] | 4.6 |
| **10** | ***780*** | 28.0 | [28.0, 28.1] | 28.0 | [28.0, 28.1] | 4.7 | 0.0 | [-0.1, 0.1] | 0.0 | [-0.1, 0.2] | 4.5 |
|  | **set mean change groups** | **average TL-BS positive** | | | | | **peak TL-BS positive** | | | | |
|  |  | **change groups** | | **control groups** | | **% sig. t-tests** | **change groups** | | **control groups** | | **% sig. t-tests** |
| **run** |  | **Mean** | **95% CI** | **Mean** | **95% CI** |  | **Mean** | **95% CI** | **Mean** | **95% CI** |  |
| **1** | ***600*** | 33.4 | [33.3, 33.5] | 33.4 | [33.3, 33.5] | 4.5 | 82.7 | [82.4, 83.0] | 82.5 | [82.2, 82.8] | 6.3 |
| **2** | ***620*** | 33.4 | [33.3, 33.5] | 33.4 | [33.3, 33.5] | 5.2 | 82.4 | [82.1, 82.7] | 82.5 | [82.3, 82.8] | 5.2 |
| **3** | ***640*** | 33.4 | [33.3, 33.5] | 33.4 | [33.3, 33.5] | 6.0 | 82.3 | [82.1, 82.6] | 82.5 | [82.2, 82.8] | 5.4 |
| **4** | ***660*** | 33.5 | [33.4, 33.6] | 33.4 | [33.3, 33.5] | 5.0 | 82.7 | [82.5, 83.0] | 82.5 | [82.3, 82.8] | 5.2 |
| **5** | ***680*** | 33.4 | [33.3, 33.5] | 33.4 | [33.3, 33.5] | 6.1 | 82.4 | [82.2, 82.7] | 82.3 | [82.0, 82.6] | 5.3 |
| **6** | ***700*** | 33.3 | [33.2, 33.4] | 33.5 | [33.4, 33.6] | 4.2 | 82.2 | [81.9, 82.5] | 82.6 | [82.4, 82.9] | 5.0 |
| **7** | ***720*** | 33.4 | [33.3, 33.5] | 33.4 | [33.3, 33.5] | 5.5 | 82.4 | [82.1, 82.7] | 82.4 | [82.1, 82.7] | 5.6 |
| **8** | ***740*** | 33.4 | [33.3, 33.5] | 33.4 | [33.3, 33.5] | 4.8 | 82.5 | [82.2, 82.8] | 82.3 | [82.0, 82.6] | 6.2 |
| **9** | ***760*** | 33.4 | [33.3, 33.5] | 33.4 | [33.3, 33.5] | 4.9 | 82.4 | [82.1, 82.7] | 82.7 | [82.4, 82.9] | 5.2 |
| **10** | ***780*** | 33.3 | [33.2, 33.4] | 33.5 | [33.4, 33.6] | 4.6 | 82.5 | [82.3, 82.8] | 82.7 | [82.4, 83.0] | 4.6 |
|  | **set mean change groups** | **average TL-BS negative** | | | | | **peak TL-BS negative** | | | | |
|  |  | **change groups** | | **control groups** | | **% sig. t-tests** | **change groups** | | **control groups** | | **% sig. t-tests** |
| **run** |  | **Mean** | **95% CI** | **Mean** | **95% CI** |  | **Mean** | **95% CI** | **Mean** | **95% CI** |  |
| **1** | ***600*** | -33.4 | [-33.5, -33.3] | -33.4 | [-33.5, -33.3] | 4.8 | -82.4 | [-82.7, -82.1] | -82.4 | [-82.7, -82.2] | 5.1 |
| **2** | ***620*** | -33.4 | [-33.5, -33.3] | -33.5 | [-33.6, -33.4] | 5.8 | -82.5 | [-82.7, -82.2] | -82.7 | [-83.0, -82.4] | 4.2 |
| **3** | ***640*** | -33.3 | [-33.4, -33.2] | -33.5 | [-33.6, -33.4] | 4.5 | -82.4 | [-82.6, -82.1] | -82.7 | [-82.9, -82.4] | 6.3 |
| **4** | ***660*** | -33.5 | [-33.6, -33.4] | -33.4 | [-33.5, -33.3] | 4.8 | -82.8 | [-83.1, -82.5] | -82.4 | [-82.7, -82.1] | 2.9 |
| **5** | ***680*** | -33.4 | [-33.5, -33.3] | -33.4 | [-33.5, -33.3] | 4.1 | -82.6 | [-82.8, -82.3] | -82.7 | [-82.9, -82.4] | 5.5 |
| **6** | ***700*** | -33.4 | [-33.5, -33.3] | -33.5 | [-33.6, -33.4] | 4.6 | -82.5 | [-82.8, -82.3] | -82.7 | [-83.0, -82.4] | 4.9 |
| **7** | ***720*** | -33.3 | [-33.4, -33.2] | -33.3 | [-33.4, -33.2] | 4.3 | -82.5 | [-82.8, -82.3] | -82.3 | [-82.6, -82.0] | 4.6 |
| **8** | ***740*** | -33.4 | [-33.5, -33.3] | -33.4 | [-33.5, -33.3] | 4.5 | -82.6 | [-82.8, -82.3] | -82.4 | [-82.6, -82.1] | 5.3 |
| **9** | ***760*** | -33.4 | [-33.5, -33.3] | -33.4 | [-33.5, -33.3] | 4.5 | -82.5 | [-82.8, -82.2] | -82.6 | [-82.8, -82.3] | 4.5 |
| **10** | ***780*** | -33.4 | [-33.5, -33.3] | -33.3 | [-33.4, -33.2] | 5.1 | -82.5 | [-82.7, -82.2] | -82.4 | [-82.7, -82.1] | 4.4 |
| *Results for the TL-BS Mean rt increasing simulation, consisting of ten runs r of 1000 ‘studies’ j, each with 52 ‘individuals’ i. ‘Set mean change groups’ is the mean value used for generating rt values for the change groups. ’% sig. t-tests’ is the percentage of studies in which t-tests for group differences return a p < .05.*  *Mean rt is kept at 600 for control groups. SD at the rt level is kept at 30 for both group types. No bias is created.* | | | | | | | | | | | |

| **Bias increasing**  ***3 points/run: 0 - 27*** | | | | | | | | | | | |
| --- | --- | --- | --- | --- | --- | --- | --- | --- | --- | --- | --- |
|  | **set bias change groups** | **TL-BS variability** | | | | | **Bias Index** | | | | |
|  |  | **change groups** | | **control groups** | | **% sig. t-tests** | **change groups** | | **control groups** | | **% sig. t-tests** |
| **run** |  | **Mean** | **95% CI** | **Mean** | **95% CI** |  | **Mean** | **95% CI** | **Mean** | **95% CI** |  |
| **1** | **0** | 28.0 | [27.9, 28.0] | 28.0 | [28.0, 28.1] | 4.4 | 0.0 | [-0.1, 0.1] | -0.1 | [-0.16, 0.07] | 5.1 |
| **2** | **3** | 28.1 | [28.0, 28.1] | 28.0 | [28.0, 28.1] | 5.0 | 3.0 | [2.9, 3.1] | 0.0 | [-0.12, 0.11] | 19.0 |
| **3** | **6** | 28.0 | [27.9, 28.1] | 28.0 | [27.9, 28.0] | 4.7 | 5.9 | [5.8, 6.0] | -0.1 | [-0.17, 0.05] | 59.3 |
| **4** | **9** | 28.0 | [28.0, 28.1] | 28.0 | [28.0, 28.1] | 4.1 | 9.0 | [8.9, 9.1] | 0.0 | [-0.13, 0.10] | 92.8 |
| **5** | **12** | 28.0 | [28.0, 28.1] | 28.1 | [28.0, 28.1] | 4.2 | 12.0 | [11.8, 12.1] | 0.0 | [-0.10, 0.13] | 99.5 |
| **6** | **15** | 28.0 | [28.0, 28.1] | 28.0 | [27.9, 28.1] | 3.7 | 14.8 | [14.7, 14.9] | -0.1 | [-0.18, 0.04] | 100 |
| **7** | **18** | 28.1 | [28.0, 28.1] | 28.0 | [28.0, 28.1] | 4.9 | 17.9 | [17.8, 18.1] | -0.1 | [-0.18, 0.05] | 100 |
| **8** | **21** | 28.0 | [27.9, 28.1] | 28.0 | [28.0, 28.1] | 4.1 | 20.8 | [20.6, 20.9] | 0.0 | [-0.15, 0.08] | 100 |
| **9** | **24** | 28.1 | [28.1, 28.2] | 28.1 | [28.0, 28.1] | 4.8 | 23.8 | [23.7, 23.9] | 0.0 | [-0.14, 0.10] | 100 |
| **10** | **27** | 28.1 | [28.0, 28.1] | 28.0 | [28.0, 28.1] | 5.2 | 26.8 | [26.7, 26.9] | 0.0 | [-0.09, 0.14] | 100 |
|  | **set bias change groups** | **average TL-BS positive** | | | | | **peak TL-BS positive** | | | | |
|  |  | **change groups** | | **control groups** | | **% sig. t-tests** | **change groups** | | **control groups** | | **% sig. t-tests** |
| **run** |  | **Mean** | **95% CI** | **Mean** | **95% CI** |  | **Mean** | **95% CI** | **Mean** | **95% CI** |  |
| **1** | **0** | 33.4 | [33.3, 33.5] | 33.4 | [33.3, 33.5] | 6.6 | 82.4 | [82.2, 82.7] | 82.5 | [82.2, 82.7] | 5.3 |
| **2** | **3** | 34.5 | [34.4, 34.6] | 33.4 | [33.3, 33.5] | 6.9 | 85.4 | [85.1, 85.7] | 82.4 | [82.1, 82.7] | 7.1 |
| **3** | **6** | 35.7 | [35.6, 35.8] | 33.4 | [33.3, 33.5] | 17.1 | 88.4 | [88.1, 88.7] | 82.3 | [82.0, 82.6] | 16.0 |
| **4** | **9** | 37.0 | [36.9, 37.1] | 33.4 | [33.3, 33.5] | 32.8 | 91.4 | [91.1, 91.7] | 82.5 | [82.3, 82.8] | 30.8 |
| **5** | **12** | 38.3 | [38.2, 38.4] | 33.5 | [33.3, 33.6] | 52.4 | 94.3 | [94.0, 94.5] | 82.6 | [82.3, 82.9] | 45.7 |
| **6** | **15** | 39.4 | [39.3, 39.5] | 33.3 | [33.2, 33.4] | 73.5 | 96.8 | [96.5, 97.1] | 82.2 | [81.9, 82.5] | 64.4 |
| **7** | **18** | 41.0 | [40.8, 41.1] | 33.4 | [33.3, 33.5] | 89.9 | 100.1 | [99.9, 100.4] | 82.4 | [82.1, 82.7] | 82.6 |
| **8** | **21** | 42.2 | [42.1, 42.3] | 33.5 | [33.4, 33.6] | 95.9 | 102.9 | [102.6, 103.1] | 82.7 | [82.4, 82.9] | 89.2 |
| **9** | **24** | 43.8 | [43.7, 43.9] | 33.5 | [33.4, 33.6] | 99.2 | 106.0 | [105.7, 106.3] | 82.6 | [82.4, 82.9] | 96.0 |
| **10** | **27** | 45.3 | [45.2, 45.4] | 33.4 | [33.3, 33.5] | 99.6 | 109.0 | [108.7, 109.3] | 82.6 | [82.4, 82.9] | 98.6 |
|  | **set bias change groups** | **average TL-BS negative** | | | | | **peak TL-BS negative** | | | | |
|  |  | **change groups** | | **control groups** | | **% sig. t-tests** | **change groups** | | **control groups** | | **% sig. t-tests** |
| **run** |  | **Mean** | **95% CI** | **Mean** | **95% CI** |  | **Mean** | **95% CI** | **Mean** | **95% CI** |  |
| **1** | **0** | -33.3 | [-33.4, -33.2] | -33.4 | [-33.5, -33.3] | 4.7 | -82.4 | [-82.7, -82.2] | -82.6 | [-82.9, -82.3] | 5.6 |
| **2** | **3** | -32.4 | [-32.5, -32.3] | -33.3 | [-33.4, -33.2] | 6.9 | -79.5 | [-79.8, -79.3] | -82.4 | [-82.7, -82.1] | 7.5 |
| **3** | **6** | -31.3 | [-31.4, -31.2] | -33.4 | [-33.5, -33.3] | 13.4 | -76.7 | [-77.0, -76.4] | -82.5 | [-82.7, -82.2] | 15.2 |
| **4** | **9** | -30.4 | [-30.5, -30.3] | -33.4 | [-33.5, -33.3] | 23.9 | -73.7 | [-74.0, -73.4] | -82.5 | [-82.7, -82.2] | 28.5 |
| **5** | **12** | -29.4 | [-29.5, -29.3] | -33.4 | [-33.5, -33.3] | 40.1 | -71.0 | [-71.2, -70.7] | -82.6 | [-82.9, -82.3] | 44.5 |
| **6** | **15** | -28.4 | [-28.5, -28.3] | -33.4 | [-33.5, -33.3] | 56.1 | -67.7 | [-67.9, -67.4] | -82.4 | [-82.7, -82.1] | 67.5 |
| **7** | **18** | -27.6 | [-27.7, -27.5] | -33.5 | [-33.6, -33.4] | 71.3 | -64.9 | [-65.1, -64.6] | -82.7 | [-83.0, -82.4] | 80.0 |
| **8** | **21** | -26.7 | [-26.8, -26.6] | -33.5 | [-33.6, -33.4] | 78.0 | -62.0 | [-62.3, -61.8] | -82.7 | [-83.0, -82.4] | 90.5 |
| **9** | **24** | -26.0 | [-26.1, -25.9] | -33.4 | [-33.5, -33.3] | 84.7 | -59.1 | [-59.3, -58.8] | -82.6 | [-82.9, -82.4] | 97.5 |
| **10** | **27** | -25.2 | [-25.3, -25.0] | -33.5 | [-33.6, -33.4] | 90.3 | -56.1 | [-56.3, -55.8] | -82.7 | [-83.0, -82.4] | 99.1 |
| *Results for the TL-BS Bias increasing simulation, consisting of ten runs r of 1000 ‘studies’ j, each with 52 ‘individuals’ i.‘Set bias change groups’ is the mean rt difference between incongruent and congruent trials implied when generating rt values for the change groups. ‘% sig. t-tests’ is the percentage of studies in which t-tests for group differences return a p < .05.*  *Mean rt is kept at 600 for control groups. SD at the rt level is kept at 30 for both group types.* | | | | | | | | | | | |

| **Dynamic bias frequency increasing**  ***bias = +\|- 20, switches: 0 - 9*** | | | | | | | | | | | |
| --- | --- | --- | --- | --- | --- | --- | --- | --- | --- | --- | --- |
|  | **n bias switch** | **TL-BS variability** | | | | | **Bias Index** | | | | |
|  |  | **change groups** | | **control groups** | | **% sig. t-tests** | **change groups** | | **control groups** | | **% sig. t-tests** |
| **run** |  | **Mean** | **95% CI** | **Mean** | **95% CI** |  | **Mean** | **95% CI** | **Mean** | **95% CI** |  |
| **1** | ***0*** | 28.0 | [28.0, 28.1] | 28.0 | [27.9, 28.1] | 5.3 | 19.8 | [19.7, 20.0] | 19.9 | [19.8, 20.00] | 3.9 |
| **2** | ***1*** | 28.2 | [28.1, 28.3] | 28.1 | [28.0, 28.1] | 5.0 | -0.1 | [-0.2, 0.0] | 19.9 | [19.8, 20.01] | 100 |
| **3** | ***2*** | 28.4 | [28.3, 28.4] | 28.0 | [28.0, 28.1] | 5.9 | 6.6 | [6.5, 6.7] | 19.9 | [19.8, 19.98] | 100 |
| **4** | ***3*** | 28.6 | [28.6, 28.7] | 28.0 | [28.0, 28.1] | 6.9 | -0.1 | [-0.2, 0.0] | 19.8 | [19.7, 19.90] | 100 |
| **5** | ***4*** | 28.8 | [28.8, 28.9] | 28.0 | [28.0, 28.1] | 8.9 | 4.2 | [4.0, 4.3] | 19.8 | [19.7, 19.93] | 100 |
| **6** | ***5*** | 29.0 | [28.9, 29.0] | 28.1 | [28.0, 28.1] | 11.2 | 0.0 | [-0.1, 0.1] | 19.9 | [19.8, 20.00] | 100 |
| **7** | ***6*** | 29.1 | [29.1, 29.2] | 28.0 | [27.9, 28.1] | 13.4 | 3.9 | [3.8, 4.1] | 19.8 | [19.6, 19.87] | 100 |
| **8** | ***7*** | 29.3 | [29.2, 29.4] | 28.0 | [28.0, 28.1] | 16.2 | -1.2 | [-1.3, -1.1] | 19.9 | [19.8, 19.99] | 100 |
| **9** | ***8*** | 29.3 | [29.3, 29.4] | 28.0 | [28.0, 28.1] | 16.1 | 3.9 | [3.8, 4.0] | 19.8 | [19.7, 19.93] | 100 |
| **10** | ***9*** | 29.5 | [29.5, 29.6] | 28.0 | [28.0, 28.1] | 19.6 | 0.0 | [-0.1, 0.1] | 19.9 | [19.8, 19.99] | 100 |
|  | **n bias switch** | **average TL-BS positive** | | | | | **peak TL-BS positive** | | | | |
|  |  | **change groups** | | **control groups** | | **% sig. t-tests** | **change groups** | | **control groups** | | **% sig. t-tests** |
| **run** |  | **Mean** | **95% CI** | **Mean** | **95% CI** |  | **Mean** | **95% CI** | **Mean** | **95% CI** |  |
| **1** | ***0*** | 41.8 | [41.7, 41.9] | 41.8 | [41.7, 41.9] | 5.3 | 102.0 | [101.8, 102.3] | 102.0 | [101.7, 102.2] | 5.8 |
| **2** | ***1*** | 37.1 | [37.0, 37.2] | 41.8 | [41.7, 41.9] | 49.1 | 90.3 | [90.1, 90.6] | 102.2 | [102.0, 102.5] | 44.8 |
| **3** | ***2*** | 38.8 | [38.7, 38.9] | 41.7 | [41.6, 41.8] | 22.8 | 94.8 | [94.5, 95.0] | 102.2 | [101.9, 102.5] | 20.6 |
| **4** | ***3*** | 37.0 | [36.9, 37.1] | 41.8 | [41.7, 41.9] | 49.9 | 89.7 | [89.5, 90.0] | 102.1 | [101.9, 102.4] | 48.0 |
| **5** | ***4*** | 38.0 | [37.8, 38.1] | 41.9 | [41.8, 42.0] | 35.5 | 92.8 | [92.5, 93.1] | 102.1 | [101.8, 102.4] | 31.0 |
| **6** | ***5*** | 36.6 | [36.5, 36.7] | 41.9 | [41.8, 42.0] | 57.6 | 89.4 | [89.1, 89.7] | 102.1 | [101.9, 102.4] | 50.3 |
| **7** | ***6*** | 37.7 | [37.6, 37.8] | 41.8 | [41.7, 41.9] | 38.9 | 92.3 | [92.1, 92.6] | 102.0 | [101.7, 102.3] | 32.2 |
| **8** | ***7*** | 36.0 | [35.9, 36.2] | 41.8 | [41.7, 41.9] | 64.6 | 88.0 | [87.7, 88.2] | 102.1 | [101.8, 102.3] | 58.1 |
| **9** | ***8*** | 37.5 | [37.4, 37.6] | 41.7 | [41.6, 41.8] | 42.6 | 91.8 | [91.5, 92.1] | 102.0 | [101.7, 102.2] | 36.6 |
| **10** | ***9*** | 36.3 | [36.2, 36.4] | 41.8 | [41.7, 41.9] | 61.8 | 88.6 | [88.3, 88.9] | 102.1 | [101.9, 102.4] | 54.5 |
|  | **n bias switch** | **average TL-BS negative** | | | | | **peak TL-BS negative** | | | | |
|  |  | **change groups** | | **control groups** | | **% sig. t-tests** | **change groups** | | **control groups** | | **% sig. t-tests** |
| **run** |  | **Mean** | **95% CI** | **Mean** | **95% CI** |  | **Mean** | **95% CI** | **Mean** | **95% CI** |  |
| **1** | ***0*** | -27.1 | [-27.2, -27.0] | -26.9 | [-27.0, -26.8] | 4.8 | -63.1 | [-63.4, -62.9] | -62.7 | [-62.9, -62.4] | 3.7 |
| **2** | ***1*** | -37.1 | [-37.2, -36.9] | -27.1 | [-27.2, -27.0] | 96.4 | -90.3 | [-90.6, -90.1] | -63.0 | [-63.3, -62.7] | 98.2 |
| **3** | ***2*** | -34.6 | [-34.7, -34.4] | -27.1 | [-27.2, -27.0] | 81.5 | -83.8 | [-84.1, -83.5] | -63.1 | [-63.3, -62.8] | 87.8 |
| **4** | ***3*** | -36.9 | [-37.0, -36.8] | -27.0 | [-27.1, -26.9] | 97.2 | -89.9 | [-90.2, -89.6] | -63.0 | [-63.3, -62.7] | 97.8 |
| **5** | ***4*** | -35.3 | [-35.5, -35.2] | -27.0 | [-27.1, -26.9] | 88.6 | -86.2 | [-86.4, -85.9] | -63.0 | [-63.3, -62.8] | 94.5 |
| **6** | ***5*** | -36.6 | [-36.7, -36.5] | -27.0 | [-27.2, -26.9] | 95.3 | -89.3 | [-89.6, -89.1] | -62.9 | [-63.2, -62.6] | 98.1 |
| **7** | ***6*** | -35.2 | [-35.3, -35.1] | -27.0 | [-27.1, -26.9] | 89.8 | -85.8 | [-86.1, -85.5] | -62.9 | [-63.2, -62.7] | 93.6 |
| **8** | ***7*** | -36.8 | [-36.9, -36.7] | -27.0 | [-27.1, -26.9] | 96.6 | -90.1 | [-90.4, -89.8] | -63.0 | [-63.2, -62.7] | 98.1 |
| **9** | ***8*** | -34.9 | [-35.0, -34.7] | -27.0 | [-27.1, -26.9] | 86.9 | -85.0 | [-85.3, -84.7] | -63.1 | [-63.4, -62.8] | 91.3 |
| **10** | ***9*** | -36.1 | [-36.2, -36.0] | -26.96 | [-27.1, -26.9] | 95.4 | -88.5 | [-88.7, -88.2] | -62.9 | [-63.1, -62.6] | 97.3 |
| *Results for the TL-BS dynamic increasing simulation, consisting of ten runs r of 1000 ‘studies’ j, each with 52 ‘individuals’ i.‘n bias switch’ is the number of times bias switches from +20 to -20 and vice versa, which happens after every 1/r trials. ‘% sig. t-tests’ is the percentage of studies in which t-tests for group differences return a p < .05.*  *Bias is kept at +20 for control groups. SD at the rt level is kept at 30 for both groups and both trial types.* | | | | | | | | | | | |

| **Dynamic bias magnitude increasing**  ***Bias sign switches = 3, bias magnitude = +\|- 20 - 46*** | | | | | | | | | | | |
| --- | --- | --- | --- | --- | --- | --- | --- | --- | --- | --- | --- |
|  | **Set bias** | **TL-BS variability** | | | | | **Bias Index** | | | | |
|  |  | **change groups** | | **control groups** | | **% sig. t-tests** | **change groups** | | **control groups** | | **% sig. t-tests** |
| **run** |  | **Mean** | **95% CI** | **Mean** | **95% CI** |  | **Mean** | **95% CI** | **Mean** | **95% CI** |  |
| **1** | ***20*** | 28.6 | [28.6, 28.7] | 28.6 | [28.6, 28.7] | 4.2 | 0.0 | [-0.2, 0.1] | -0.1 | [-0.2, 0.1] | 4.8 |
| **2** | ***23*** | 28.8 | [28.8, 28.9] | 28.6 | [28.6, 28.7] | 4.8 | 0.0 | [-0.1, 0.1] | 0.0 | [-0.1, 0.1] | 4.7 |
| **3** | ***26*** | 29.0 | [29.0, 29.1] | 28.6 | [28.6, 28.7] | 6.2 | 0.0 | [-0.1, 0.1] | -0.1 | [-0.2, 0.0] | 5.4 |
| **4** | ***29*** | 29.2 | [29.2, 29.3] | 28.6 | [28.6, 28.7] | 7.2 | 0.0 | [-0.1, 0.1] | -0.1 | [-0.2, 0.0] | 4.7 |
| **5** | ***32*** | 29.5 | [29.4, 29.5] | 28.6 | [28.6, 28.7] | 10.1 | 0.0 | [-0.1, 0.1] | -0.1 | [-0.2, 0.1] | 5.2 |
| **6** | ***35*** | 29.8 | [29.7, 29.8] | 28.6 | [28.6, 28.7] | 12.9 | 0.0 | [-0.1, 0.1] | 0.1 | [-0.1, 0.2] | 5.7 |
| **7** | ***38*** | 30.1 | [30.0, 30.1] | 28.7 | [28.6, 28.7] | 20.3 | 0.1 | [0.0, 0.2] | -0.1 | [-0.2, 0.0] | 5.3 |
| **8** | ***41*** | 30.3 | [30.3, 30.4] | 28.6 | [28.6, 28.7] | 24.5 | 0.1 | [-0.1, 0.2] | 0.0 | [-0.2, 0.1] | 5.4 |
| **9** | ***44*** | 30.7 | [30.6, 30.7] | 28.6 | [28.5, 28.6] | 34.8 | 0.0 | [-0.1, 0.2] | 0.0 | [-0.1, 0.1] | 5.2 |
| **10** | ***47*** | 31.0 | [30.9, 31.0] | 28.6 | [28.6, 28.7] | 42.7 | -0.1 | [-0.2, 0.1] | -0.1 | [-0.2, 0.0] | 5.1 |
|  | **Set bias** | **average TL-BS positive** | | | | | **peak TL-BS positive** | | | | |
|  |  | **change groups** | | **control groups** | | **% sig. t-tests** | **change groups** | | **control groups** | | **% sig. t-tests** |
| **run** |  | **Mean** | **95% CI** | **Mean** | **95% CI** |  | **Mean** | **95% CI** | **Mean** | **95% CI** |  |
| **1** | ***20*** | 36.9 | [36.8, 37.05] | 36.87 | [36.76, 36.98] | 4.7 | 89.8 | [89.5, 90.1] | 90.0 | [89.7, 90.3] | 5.3 |
| **2** | ***23*** | 38.0 | [37.9, 38.11] | 36.83 | [36.72, 36.94] | 7.3 | 92.1 | [91.8, 92.4] | 89.9 | [89.6, 90.1] | 5.3 |
| **3** | ***26*** | 39.3 | [39.2, 39.41] | 36.94 | [36.83, 37.05] | 14.5 | 94.5 | [94.2, 94.8] | 90.0 | [89.7, 90.3] | 9.9 |
| **4** | ***29*** | 40.6 | [40.4, 40.68] | 36.90 | [36.79, 37.01] | 28.5 | 96.9 | [96.6, 97.2] | 89.9 | [89.6, 90.1] | 18.1 |
| **5** | ***32*** | 42.0 | [41.9, 42.11] | 36.77 | [36.66, 36.88] | 47.9 | 99.4 | [99.1, 99.7] | 89.7 | [89.4, 90.0] | 30.7 |
| **6** | ***35*** | 43.5 | [43.4, 43.62] | 36.91 | [36.80, 37.02] | 65.3 | 101.9 | [101.6, 102.2] | 89.8 | [89.5, 90.1] | 41.5 |
| **7** | ***38*** | 45.3 | [45.2, 45.46] | 36.89 | [36.77, 37.00] | 84.5 | 104.9 | [104.6, 105.2] | 90.0 | [89.7, 90.3] | 58.4 |
| **8** | ***41*** | 47.1 | [46.9, 47.19] | 36.90 | [36.79, 37.01] | 94.9 | 107.6 | [107.3, 108.0] | 89.7 | [89.5, 90.0] | 72.3 |
| **9** | ***44*** | 49.0 | [48.8, 49.09] | 36.84 | [36.73, 36.95] | 99.2 | 110.5 | [110.2, 110.9] | 89.8 | [89.5, 90.1] | 83.9 |
| **10** | ***47*** | 51.0 | [50.8, 51.12] | 36.81 | [36.70, 36.92] | 99.8 | 113.5 | [113.2, 113.8] | 89.8 | [89.5, 90.1] | 92.6 |
|  | **Set bias** | **average TL-BS negative** | | | | | **peak TL-BS negative** | | | | |
|  |  | **change groups** | | **control groups** | | **% sig. t-tests** | **change groups** | | **control groups** | | **% sig. t-tests** |
| **run** |  | **Mean** | **95% CI** | **Mean** | **95% CI** |  | **Mean** | **95% CI** | **Mean** | **95% CI** |  |
| **1** | ***20*** | -36.9 | [-37.0, -36.7] | -36.8 | [-36.9, -36.7] | 4.3 | -89.7 | [-90.0, -89.5] | -90.0 | [-90.3, -89.7] | 4.7 |
| **2** | ***23*** | -37.9 | [-38.0, -37.8] | -36.8 | [-36.9, -36.7] | 7.4 | -91.9 | [-92.2, -91.6] | -89.7 | [-90.0, -89.5] | 5.1 |
| **3** | ***26*** | -39.1 | [-39.3, -39.0] | -36.8 | [-36.9, -36.7] | 14.2 | -94.3 | [-94.6, -94.0] | -89.8 | [-90.1, -89.5] | 10.9 |
| **4** | ***29*** | -40.5 | [-40.6, -40.4] | -36.9 | [-37.0, -36.8] | 29.5 | -96.8 | [-97.1, -96.5] | -90.0 | [-90.3, -89.7] | 16.6 |
| **5** | ***32*** | -41.8 | [-41.9, -41.6] | -36.9 | [-37.0, -36.7] | 43.1 | -98.9 | [-99.2, -98.6] | -89.9 | [-90.2, -89.6] | 26.6 |
| **6** | ***35*** | -43.4 | [-43.6, -43.3] | -36.8 | [-36.9, -36.7] | 66.4 | -101.6 | [-101.9, -101.3] | -89.9 | [-90.2, -89.6] | 39.2 |
| **7** | ***38*** | -45.2 | [-45.3, -45.1] | -36.8 | [-36.9, -36.7] | 84.9 | -104.5 | [-104.9, -104.2] | -89.9 | [-90.2, -89.7] | 55.1 |
| **8** | ***41*** | -46.8 | [-47.0, -46.7] | -36.8 | [-36.9, -36.7] | 93.0 | -107.2 | [-107.5, -106.9] | -89.9 | [-90.2, -89.6] | 69.7 |
| **9** | ***44*** | -48.9 | [-49.0, -48.7] | -36.9 | [-37.0, -36.8] | 98.7 | -110.5 | [-110.8, -110.2] | -89.7 | [-90.0, -89.5] | 85.5 |
| **10** | ***47*** | -50.8 | [-51.0, -50.7] | -36.8 | [-36.9, -36.7] | 99.7 | -113.2 | [-113.6, -112.9] | -89.7 | [-90.0, -89.4] | 93.4 |
| *Results for the TL-BS dynamic increasing simulation, consisting of ten runs r of 1000 ‘studies’ j, each with 52 ‘individuals’ i.‘. Set bias is the magnitude of bias in the change groups. Bias switches sign (f.i. +20 to-20 and v.v.) three times in all datasets (change and control groups). ‘% sig. t-tests’ is the percentage of studies in which t-tests for group differences return a p < .05.*  *Bias magnitude is kept at +\|- 20 for control groups. SD at the rt level is kept at 30 for both groups and both trial types* | | | | | | | | | | | |
